# Indoor Organic Photovoltaics with Over 29% Efficiency and Great Stability Enabled by Giant Dimeric Acceptors with Hypsochromic Absorption and High Glass Transition Temperature

**DOI:** 10.1002/advs.202512690

**Published:** 2025-10-15

**Authors:** Bosen Zou, Ho Ming Ng, Zhengkai Li, Yan Wang, Qingyuan Wang, Dezhang Chen, Zefan Yao, Hongxiang Li, Chunliang Li, Xianghao Zeng, Wei Liu, Jonathan E. Halpert, Huawei Hu, Chunhui Duan, Zonglong Zhu, Tom Wu, Wai‐Yeung Wong, Zhi‐Guo Zhang, He Yan, Han Yu

**Affiliations:** ^1^ State Key Laboratory of Organic/Inorganic Composites Beijing Advanced Innovation Center for Soft Matter Science and Engineering Beijing University of Chemical Technology Beijing 100029 China; ^2^ Department of Chemistry and Hong Kong Branch of Chinese National Engineering Research Center for Tissue Restoration and Reconstruction The Hong Kong University of Science and Technology Hong Kong 999077 China; ^3^ Department of Chemistry and Hong Kong Institute for Clean Energy City University of Hong Kong Hong Kong 999077 China; ^4^ Department of Chemistry The Hong Kong University of Science and Technology Hong Kong 999077 China; ^5^ College of Chemistry and Molecular Engineering Peking University Beijing 100029 China; ^6^ College of Polymer Science and Engineering State Key Laboratory of Polymer Materials Engineering Sichuan University Chengdu 610106 China; ^7^ State Key Laboratory for Modification of Chemical Fibers and Polymer Materials College of Materials Science and Engineering Donghua University Shanghai 201620 China; ^8^ State Key Laboratory of Luminescent Materials and DevicesGuangdong Basic Research Center of Excellence for Energy & Information Polymer Materials South China University of Technology Guangzhou 510640 China; ^9^ Department of Applied Physics The Hong Kong Polytechnic University Hong Kong 999077 China; ^10^ Department of Applied Biology and Chemical Technology and Research Institute for Smart Energy The Hong Kong Polytechnic University Hong Kong 999077 China

**Keywords:** giant dimeric acceptors, high glass transition temperature, hypsochromic absorption, indoor photovoltaics, operation and mechanical stability

## Abstract

Indoor organic photovoltaics (IOPVs) are an emerging LED light recycling technology with promising applications such as indoor off‐grid ecosystem for the Internet of Things. However, efficient and stable IOPVs based on giant dimeric acceptors (GDAs) are rarely reported due to the dearth of GDAs with hypsochromic absorption (absorption onset < 850 nm) and good crystallinity. Herein, two hypsochromic GDAs are proposed with different fluorination degrees, namely DY4FO‐V and DY6FO‐V, and process a systematic study of hypsochromic acceptor materials from the small molecule to dimers and polymer. Interestingly, both hypsochromic GDAs possess better crystallinity, thus faster carrier transport and suppress recombination than small‐molecule and polymer acceptor‐based devices. With extra fluorination, PM6:DY6FO‐V exhibits higher external quantum efficiency response and tighter packing compared with PM6:DY4FO‐V. As a result, PM6:DY6FO‐V delivers a champion efficiency over 29% under a LED illumination of 2000 lux (2600 k), positioning it the highest values for GDA‐based IOPVs. Meanwhile, the high glass transition temperature of DY6FO‐V endowed corresponding devices with great photostability and enhanced mechanical stability in flexible devices, demonstrating the feasibility of practical applications of the DY6FO‐V‐based IOPVs. This research underscores the huge potential of developing hypsochromic GDAs for highly efficient IOPVs with superior stability.

## Graphical abstract

Through a systematical investigation of structure‐performance relationship from the small‐molecule acceptor to giant molecule acceptors (GDAs) and polymer acceptor, a fluorinated GDA is reported with hypsochromic absorption and high glass transition temperature. The resulting devices demonstrate a remarkable efficiency of 29.1% under indoor conditions, which emphasize the crucial role of GDA strategy for Internet of Things applications.

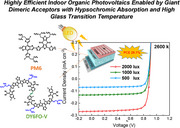

## 1 Introduction

The widespread applications of the Internet of Things (IoTs) in manufacturing, agriculture, transportation, healthcare are driving the industrial restructuring and intelligent upgrading of industries, enhancing production efficiency and product quality.^[^ ^1^, ^2^, ^3^ ^]^ Indoor organic photovoltaics (IOPVs), as a suitable alternative for IoT's energy supplier, have achieved prominent power conversion efficiencies (PCEs) ≈ 30% under the light‐emitting diodes (LED) illumination in recent years.^[^ ^4^, ^5^, ^6^ ^]^ Currently, most of these cutting‐edge efficiencies have been achieved by the conventional combination of conjugated polymer donors (PDs) and small‐molecule acceptors (SMAs). However, a critical limitation of PDs:SMAs‐typed IOPVs is their unsatisfactory long‐term stability caused by rapid diffusion properties of SMAs with intrinsically low glass transition temperatures (*T*_g_s), thus facile degradation of blend morphology.^[^ ^7^, ^8^ ^]^ To address the above‐mentioned dilemmas, acceptors with higher *T*_g_s and superior crystallinity need to be further developed, aiming to explore the next generation of high‐performance IOPVs with superior device stability.

The exploration of hypsochromic acceptors with large molecular weight (*M*_W_) is crucial for achieving stable IOPVs. Recently, our team reported a highly efficient hypsochromic polymer acceptor (PYFO‐V) with a high *T*_g_ through the synergistic effects of side chain alkoxylation^[^ ^9^, ^10^, ^11^ ^]^ and vinylene linkage,^[^ ^12^, ^13^ ^]^ raising the PCEs of indoor all‐polymer solar cells (all‐PSCs) to a level of ∼27% with remarkable device stability.^[^ ^14^ ^]^ However, the uncertain reproducibility of polymer acceptors (PAs)^[^ ^15^, ^16^, ^17^ ^]^ compared to acceptors with well‐defined chemical structure is inevitably constraining the commercialization of indoor all‐PSCs. To overcome this, giant molecule acceptor‐based OPVs,^[^ ^8^, ^18^, ^19^, ^20^, ^21^, ^22^, ^23^ ^]^ adopting acceptors with high and accurate molecular weight, are able to balance the device efficiency and stability by offering comparable crystallinity for charge transport and providing a supramolecular structure with high glass transition temperature (*T*_g_) and slow diffusion rates. However, previous IOPVs based on giant molecule acceptors entirely relied on conventional perylene diimides derivatives^[^ ^24^, ^25^, ^26^ ^]^ which suffered from several intrinsic drawbacks, including low external quantum efficiency (EQE) response in the visible range, unfavorable phase segregation, and ineffective charge transfer, thus limiting its progress. Benefitted from unremitted efforts in Y‐series SMAs^[^ ^27^, ^28^, ^29^, ^30^, ^31^, ^32^ ^]^ and SMA dimerization strategy,^[^ ^33^, ^34^, ^35^, ^36^, ^37^ ^]^ the exploration of hypsochromic Y‐series giant dimeric acceptors (GDAs) should be promising for developing IOPVs with superior efficiency and stability.

Herein, we process a BF_3_∙OEt_2_‐catalyzed Knoevenagel condensation^[^ ^38^ ^]^ to construct GDAs. This route can be considered as an alternative pathway toward GDAs compared to the traditional asymmetric monomer synthesized by pyridine‐catalyzed Knoevenagel condensation^[^ ^39^, ^40^, ^41^, ^42^ ^]^ plus palladium‐catalyzed Stille‐coupling to produce GDAs. Through connecting two alkoxy Y‐series monomers with one rigid vinylene linker, two hypsochromic GDAs, namely DY4FO‐V and DY6FO‐V, featuring with zero and mono fluorination on central end groups, were rationally synthesized for simultaneously enhancing the efficiency and stability of IOPVs. Both new GDAs yielded high open‐circuit voltage (*V*_OC_) of 1.00 V in the PM6:GDA‐based devices under one‐sun illumination, representing the highest *V*_OC_s among the binary OPVs based on Y‐series GDAs (Table S1, Supporting Information). Surprisingly, the DY6FO‐V‐based devices obtained a decent efficiency of 16.6%, with a high EQE response in the visible range and less trap‐assisted recombination. As a result, the optimized DY6FO‐V‐based device realized an inspiring PCE of 29.1% under a 2600 k LED light, marking a cutting‐edge performance for binary IOPVs. (Table S2, Supporting Information). Furthermore, the utilization of DY6FO‐V with high glass transition temperature (*T*_g_) endowed corresponding devices with outstanding operation lifetime, maintaining≈81% of the original values over 1100 h, thus highlighting the feasibility of long‐term commercial implementation. Meanwhile, the DY6FO‐V‐based‐flexible device exhibited enhanced mechanical stability compared with the small molecule acceptor‐based‐flexible device, demonstrating the huge potential of DY6FO‐V in the flexible fabrication. This study provides important guidelines for the design of hypsochromic GDAs with scalable potential in achieving IOPVs with high performance and stability.

## 2 Results and Discussion

### 2.1 Material Synthesis and Characterizations

**Figure 1:**
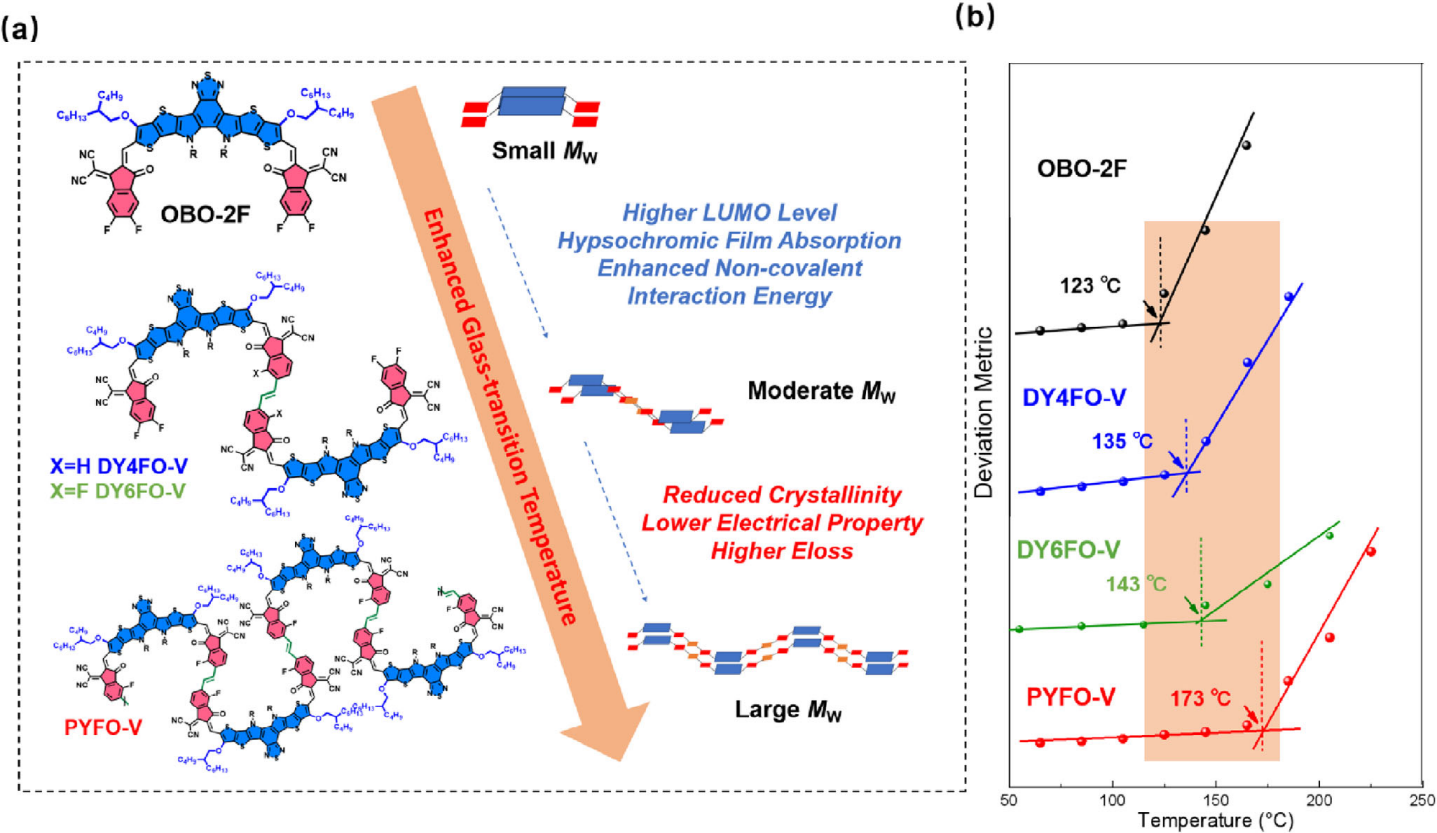
a) The design strategy of the acceptors with different *M*_W_s, where R is 2‐OD. b) The evolution of the glass transition temperature from the SMA to GDAs and to the PA.

We synthesized a series of hypsochromic acceptors, named OBO‐2F, DY4FO‐V, DY6FO‐V and PYFO‐V, based on alkoxy Y‐series monomers connected by vinylene linkers. The molecular structure of these acceptors with different molecular weights are shown in **Figure** **1**a and Scheme S1 (Supporting Information), and detailed characterizations are provided in Figures S1–S6 (Supporting Information). The thermal stability of these acceptors was investigated through thermogravimetric analysis measurement as shown in Figure S7 (Supporting Information). All these alkoxy acceptors demonstrated great thermal stability with decomposition temperature (*T*_d_, 5% weight loss) over 305 °C. In addition to *T*_d_, we conducted the deviation metric (DMT) method^[^ ^7^, ^29^ ^]^ (Figure S8, Supporting Information) to estimate their glass transition temperatures (*T*_g_s). Notably, the *T*_g_ value progressively increases with the gradually enlarged molecular weight (Figure 1b). These results suggest that, with the larger molecular size of acceptors, the diffusion rate of acceptors can be effectively inhibited, thus leading to better device stability.

**Figure 2:**
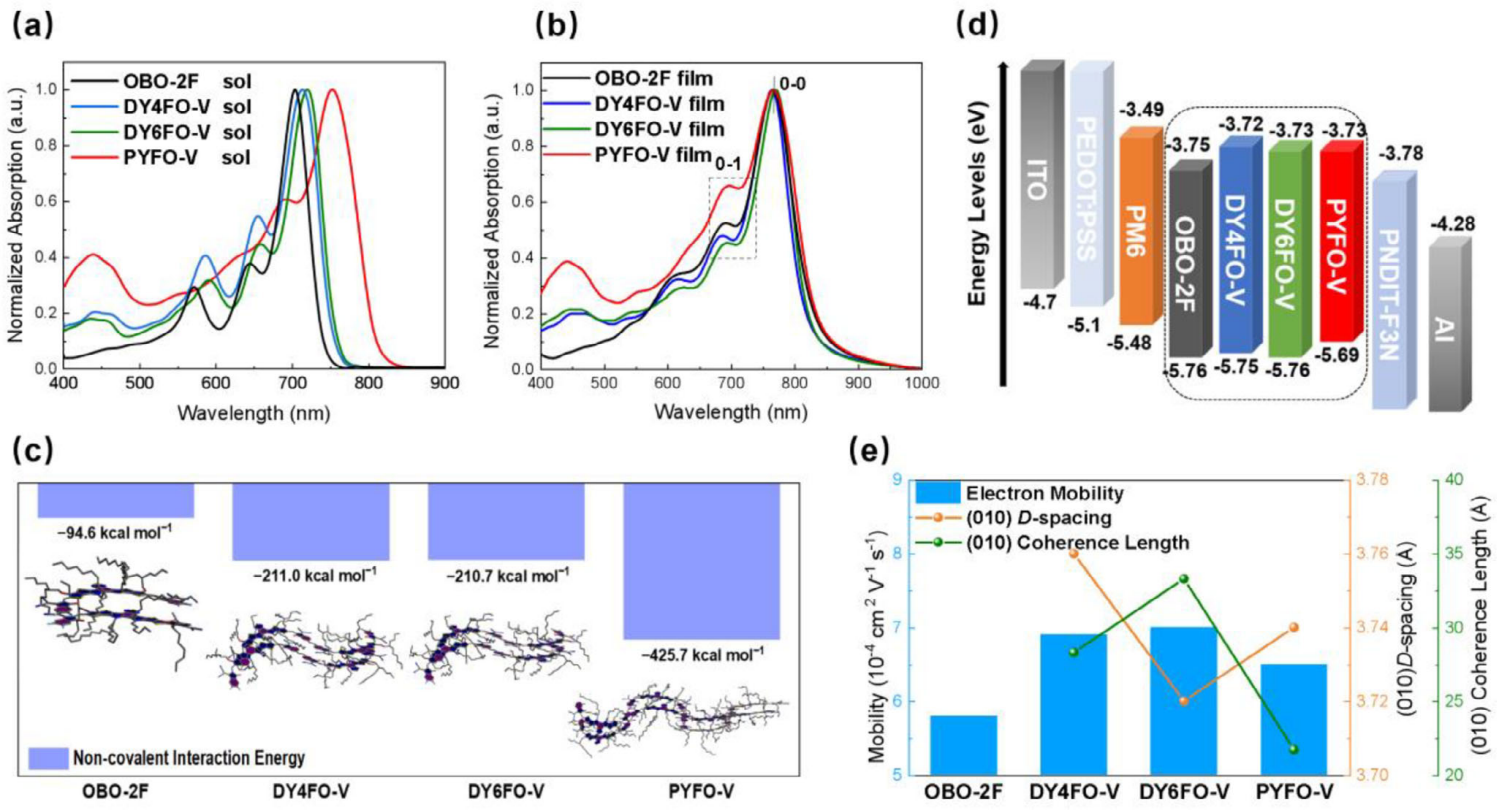
Normalized UV–vis absorption spectra of these acceptors in (a) chloroform solutions and (b) thin‐film states. c) The calculated non‐covalent interaction energy for these acceptors. d) The energy alignment of the materials and the device structure in OPVs. e) Fitted GIWAXS parameters for OOP directional *π–π* stacking peaks and electron mobility of the pure acceptors.

Subsequently, the optical properties of these acceptors in both chloroform solutions and thin‐film states were studied through UV–vis absorption spectroscopy as shown in **Figure** **2**a,b. In the solution, the absorption peak wavelength (*λ*_max_) of OBO‐2F is 704 nm, while the absorption peaks of the GDAs and PA are significantly red‐shifted, which could be owing to the longer conjugated structure of the GDAs and PA. As for the GDAs, DY4FO‐V displays a maximum absorption peak (*λ*_max,sol_) at 714 nm, while the DY6FO‐V exhibit a bathochromic *λ*_max,sol_ at 720 nm, which is attributed to the stronger intramolecular charge transfer (ICT) effect of the additional fluorination.^[^ ^43^, ^44^ ^]^ In the thin‐film state, all acceptors exhibit bathochromic absorption relative to their solution state. The corresponding optical bandgaps (*E*_g_) of OBO‐2F, DY4FO‐V, DY6FO‐V and PYFO‐V in thin‐film states are 1.49, 1.51, 1.50, and 1.48 eV, respectively, determined from their absorption onsets (*λ*_onset,film_) mentioned in the **Table** **1**. It is noted that both GDAs possess enlarged optical bandgaps compared to small‐molecule and polymeric acceptors, which could help to reduce spectral mismatch under indoor condition. In addition, the intensity ratio between the 0–0 and 0–1 vibrational transition (*I*_0–0_/*I*_0–1_) are 1.93, 2.16, 2.25, and 1.48 for OBO‐2F, DY4FO‐V, DY6FO‐V and PYFO‐V, respectively. The highest *I*_0–0_/*I*_0–1_ of DY6FO‐V suggests a more *J*‐aggregation characteristics, which is conducive to facilitating exciton generation and charge transport.^[^ ^45^ ^]^ In addition to the pure films, the UV–vis absorption spectra of the blend films are shown in Figure S9 (Supporting Information), which are matching with indoor illumination sources, beneficial for effective visible light harvesting.

**Table 1:** Optical and electrochemical properties of OBO‐2F, DY4FO‐V, DY6FO‐V, and PYFO‐V.

Material	*λ*_max,sol_ [nm]	*λ*_max,film_ [nm]	*λ*_onset,film_ [nm]	*E*_g_ ^a)^ [eV]	[*I*_0–0_/*I*_0–1_]	HOMO/LUMO^b)^ [eV]
OBO‐2F	704	766	834	1.49	1.93	−5.76/‐3.75
DY4FO‐V	714	764	819	1.51	2.16	−5.75/−3.72
DY6FO‐V PYFO‐V	720 752	771 767	825 837	1.50 1.48	2.25 1.54	−5.76/−3.73 −5.69/−3.73

^a)^ Calculated from the absorption onset of the films;

^b)^ Estimated from the reduction/oxidation onset of the CV curves.

We conducted all‐atom molecular dynamics (AA‐MD) simulations^[^ ^46^ ^]^ (Figure 2c) on these alkoxy acceptors to explore the assembly deduction. It's clearly that PYFO‐V bi‐molecule delivers a non‐covalent interaction energy (*E*_i_) of ‐425.7 kcal mol^−1^, obviously higher than that of ‐94.6 kcal mol^−1^ for OBO‐2F bi‐molecule and −210/−211 kcal mol^−1^ for GDAs bi‐molecule, confirming that the increasement of molecular weight can induce stronger non‐covalent intermolecular interactions. Enhanced non‐covalent interaction energy could benefit optimized and stabilized morphology. Besides, the electrostatic potentials (ESP) results of GDAs are shown in Figure S10 (Supporting Information). As the incorporation of fluorine atom at the linking end group moieties, the ESP becomes more negative from DY4FO‐V to DY6FO‐V, beneficial for the charge separation and electron delocalization in photovoltaic processes.^[^ ^44^ ^]^ Next, the electrochemical properties of these alkoxy acceptors were further evaluated by cyclic voltammetry (Figure S11, Supporting Information), with the energy alignment of these alkoxy acceptors, together with the classical donor PM6 (Figure S12, Supporting Information) shown in Figure 2d. The lowest unoccupied molecular orbital (LUMO)/ highest occupied molecular orbital (HOMO) levels were determined to be −5.76/−3.75, −5.75/−3.72, −5.76/−3.73 and −5.69/−3.73 eV for OBO‐2F, DY4FO‐V, DY6FO‐V and PYFO‐V, respectively, consistent with the trend of the optical bandgaps (*E*_g_s) of these acceptors. The multi‐fluorinated DY6FO‐V exhibited down‐shifted energy levels compared to its counterpart, DY4FO‐V, which is also supported by the density functional theory calculation results (Figure S13, Supporting Information).

The crystalline properties of these acceptors in the neat film state were studied using grazing‐incidence wide angle X‐ray scattering (GIWAXS). The 2D GIWAXS patterns and corresponding line cuts along the in‐plane (IP) and out‐of‐plane (OOP) directions are shown in Figures S14 and S15 (Supporting Information), with fitting parameters for OOP directional 𝜋–𝜋 stacking peaks of pure acceptors presented in Figure 2e. From the 2D patterns, OBO‐2F preferred an edge‐on molecular packing orientation in the film state. In contrast, both GDAs and the PA exhibit dominated face‐on orientation. The (010) peaks for DY4FO‐V, DY6FO‐V, and PYFO‐V films are located at 1.67, 1.69, and 1.68 Å^−1^, respectively, corresponding to the 𝜋–𝜋 stacking distance (*D*‐spacing) of 3.76, 3.72, and 3.74 Å, respectively, demonstrating a denser packing mode can be realized as the introduction of F···H non‐covalent interactions^[^ ^43^, ^47^ ^]^ between the hydrogen on the vinylene linker and the fluorine on the ending moiety of monomer blocks. Moreover, DY6FO‐V shows a slightly larger coherence length (CCL) than DY4FO‐V and PYFO‐V, indicative of a stronger crystallinity of DY6FO‐V as well. As a result, DY6FO‐V possesses a higher electron mobility (*µ*_e_) (Figure S16; Table S4, Supporting Information) of 7.0 × 10^−4^ cm^2^ V^−1^ s^−1^ compared to the other three acceptors due to the enhanced packing benefited from the better crystallinity.

### 2.2 Outdoor Photovoltaic Performance

**Figure 3:**
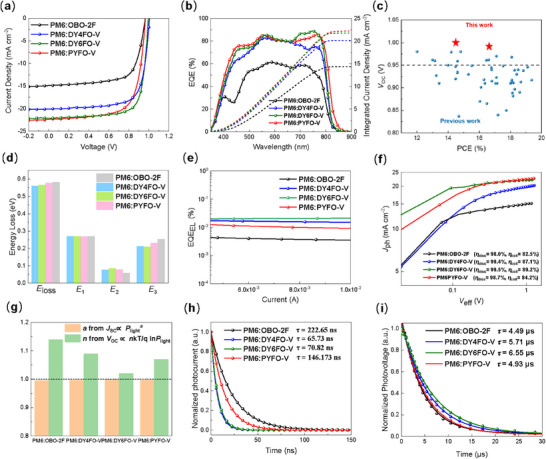
a) *J–V* curves of the optimal OPVs based on PM6:acceptors. b) Corresponding EQE spectra. c) *V*_OC_ versus PCE of OPVs based on GDAs. d) Histograms of different parts of energy loss in these devices. e) EQE_EL_ results. f) *J*_ph_ versus *V*_eff_ of the optimized devices. g) Charge carrier recombination behaviors obtained from the dependences of *J*_SC_/*V*_OC_ on light intensity. h) TPC and i) TPV results.

The photovoltaic performance of these hypsochromic acceptors under AM 1.5 G (100 mW cm^−2^) illumination was evaluated by fabricating devices based on a conventional device structure of ITO/PEDOT:PSS/PM6:Acceptor/PDINN/Al. The current density versus voltage (*J‐V*) characteristics of the optimized devices are shown in **Figure** **3**a, with specific parameters summarized in **Table** **2**. The PYFO‐V‐based devices yielded a decent PCE of 15.2% with a *V*_OC_ of 0.966 V, a short‐circuit current density (*J*_SC_) of 22.4 mA cm^−2^ and an FF of 70.1%, which is comparable to the previous report.^[^ ^14^ ^]^ Notably, both DY4FO‐V‐ and DY6FO‐V‐based devices yielded *V*_OC_s as high as 1.00 V, ranking the highest *V*_OC_s reported for binary devices based on Y‐series GDAs (Figure 3c). The optimized device based on PM6:DY6FO‐V exhibits a superior PCE of 16.6% with a *V*_OC_ of 0.994 V, a *J*_SC_ of 22.1 mA cm^−2^, and an FF of 75.5%, which is much better than the other devices. The decreased *J*_SC_s of GDA‐based OPVs should be partially derived from the blue‐shifted absorption compared to PYFO‐V. The *J*_SC_s of these devices can be verified by the external quantum efficiency (EQE) spectra (Figure 3b). The EQE curve for the PM6:DY6FO‐V device demonstrates a better overlap with LED emission spectra, accompanied by a higher EQE intensity in this range, indicating effective visible photon harvesting and efficient carrier generation. Besides, the EQE results also reveal that the increasement of *J*_SC_ from DY4FO‐V to DY6FO‐V is caused by the higher EQE contribution in the range of 700–770 nm.

**Table 2:** Photovoltaic parameters of the devices under AM 1.5 G illumination at 100 mW cm^−2^.

Active layer	*V*_OC_ [V]	*J*_SC_/*J*_cal_ [mA cm^−2^]	FF [%]	PCE [%]
PM6:OBO‐2F	0.970	14.8/14.4	66.7	9.58 (9.4 ± 0.2)
PM6:DY4FO‐V	1.007	20.1/20.2	73.0	14.7 (14.4 ± 0.2)
PM6:DY6FO‐V	0.994	22.1/21.8	75.5	16.6 (16.4 ± 0.2)
PM6:PYFO‐V	0.966	22.4/22.1	70.1	15.2 (14.9 ± 0.3)

The brackets contain averages and standard errors of PCEs based on 10 devices.

The *E*_loss_ measurements were conducted to explain the origin of high *V*_OC_s of these two GDA‐based devices. The detailed description of the experiments is shown in the methods part and the results are displayed in Figure 3d, Figure S17 and Table S5 (Supporting Information). According to the *E*_loss_ measurement, these devices performed similar radiative energy loss (Δ*E*_1_). And the Δ*E*_2_ values are 0.058 eV for the OBO‐2F‐ based device, 0.077 eV for the DY4FO‐V‐based device, 0.085 eV for the DY6FO‐V‐based device and 0.078 eV for the PYFO‐V‐based device. To measure non‐radiative recombination loss (Δ*E*_3_), we gauged electroluminescence quantum efficiency (EQE_EL_) as depicted in Figure 3e. Remarkably, the EQE_EL_ of GDA‐based devices, especially the PM6:DY6FO‐V‐based device, exhibited the highest EQE_EL_ value, leading to the smallest Δ*E*_3_ of 0.21 eV compared to the other devices. The smaller Δ*E*_3_ of DY6FO‐V‐based devices can be attributed to the fluorine‐induced conformational locking with suppressed vibrational states in the blend of PM6:DY6FO‐V,^[^ ^43^ ^]^ therefore suppressed non‐radiative recombination. The total *E*_loss_s were determined to be 0.582, 0.560, 0.565, and 0.578 eV for PM6:OBO‐2F, PM6:DY4FO‐V, PM6:DY6FO‐V, and PM6:PYFO‐V devices, respectively. The reduced *E*_loss_s of the GDA‐based devices should mainly result from the higher degree of conformational order of the GDAs, with denser packing, fewer vibration states, and suppressed non‐radiative recombination. Therefore, the reduced *E*_loss_s of these two GDA‐based devices lead to more obvious increasement of *V*_OC_s, compared to the SMA‐ and PA‐based devices.

### 2.3 Device Physics and Film Formation Kinetics

The exciton dissociation, charge transport, and recombination characteristics in the blend films were investigated to further explore the reason of different photovoltaic performances. Photocurrent density (*J*_ph_) versus effective voltage (*V*_eff_) curves are plotted in Figure 3f and the corresponding parameters presented in Table S6 (Supporting Information). For both DY4FO‐V‐ and DY6FO‐V‐based devices, the 𝜂_diss_ / 𝜂_coll_ values are significantly enhanced compared with those of the PM6:OBO‐2F‐ and PM6:PYFO‐V‐based devices, demonstrating a more efficient exciton dissociation of the GDAs‐based devices. Besides, Photoluminescence (PL) quenching experiments of these pure and blend films were further measured to study the efficiency of exciton dissociation. The corresponding PL spectra excited at 785 nm are shown in Figure S18 (Supporting Information). The PL quenching efficiency of the PM6:DY6FO‐V blend (90.0%) is higher than PM6:OBO‐2F (89.8%), PM6:DY4FO‐V (87.1%) and PM6:PYFO‐V (76.2%), revealing a more efficient charge transfer between the donor and acceptor in the blend of PM6:DY6FO‐V. Moreover, the charge recombination mechanisms of these devices were investigated by analyzing the relationship between light intensity and *V*_OC_/*J*_SC_, as depicted in Figure 3g and Figure S19 (Supporting Information). Both *V*_OC_ and *J*_SC_ versus light intensity curves show that PM6:DY6FO‐V has the least trap‐assisted and bimolecular recombination than the other devices. Considering the trap‐assisted recombination becomes a dominate factor under dim light,^[^ ^48^ ^]^ the lowest degree of trap‐assisted recombination in the DY6FO‐V‐based devices plays an important role in achieving high FF values under dim light for IOPVs.

The transient photocurrent (TPC) and transient photovoltage (TPV) measurements were employed to further investigate the charge extraction and recombination process of these devices. Figure 3h presents the TPC measurements and charge extraction times (*τ*_TPC_). The DY4FO‐V‐ and DY6FO‐V‐based blends show shorter *τ*_TPC_s, than the PM6:OBO‐2F and PM6:PYFO‐V. This result implies that the GDA‐based devices can sweep out charge rapidly, suggesting a superior charge extraction capacity. Besides, The charge carrier lifetimes (*τ*_TPV_s) from TPV measurements (Figure 3i) show the sequence of PM6:DY6FO‐V (6.55 µs) > PM6:DY4FO‐V (5.71 µs) > PM6:PYFO‐V (4.93 µs) > PM6:OBO‐2F (4.49 µs). The longest carrier lifetime of PM6:DY6FO‐V demonstrates that the DY6FO‐V‐based device possesses a slower charge recombination rate. All these device physical characterizations demonstrate that the GDA‐based devices have multiple advantages over the SMA‐ or PA‐ based ones in terms of efficient charge dissociation, suppressed charge recombination, and faster charge extraction.

To better understand the film‐formation process, in situ monitoring of the film‐drying process using techniques of time‐sensitive absorption was applied to explore the phase transition from the solution state to the solid state. The original in situ UV–vis absorption spectra are shown in Figure S20a (Supporting Information). The evolution of the peak position based on the corresponding blends were depicted in Figure S20b (Supporting Information). With the removal of chloroform, the blend films of PM6:OBO‐2F and two PM6:GDAs show similar times for the film to be solidified, which are slower than PM6:PYFO‐V. After this process, the position, shape, and intensity of the peaks do not change, indicating that molecular ordered stacking has occurred, and the acceptor has transformed into a solid phase.^[^ ^49^ ^]^ Therefore, an extra time window is given to the active blend formation of PM6:GDAs, leading molecules in the active layer to stack more orderly, evidenced by the steeper EQE band tail (Figure 3b), as well as the higher *V*_OC_ for the PM6:GDAs‐ based devices compared with the PM6:PYFO‐V ones.

### 2.4 Morphology Analysis

**Figure 4:**
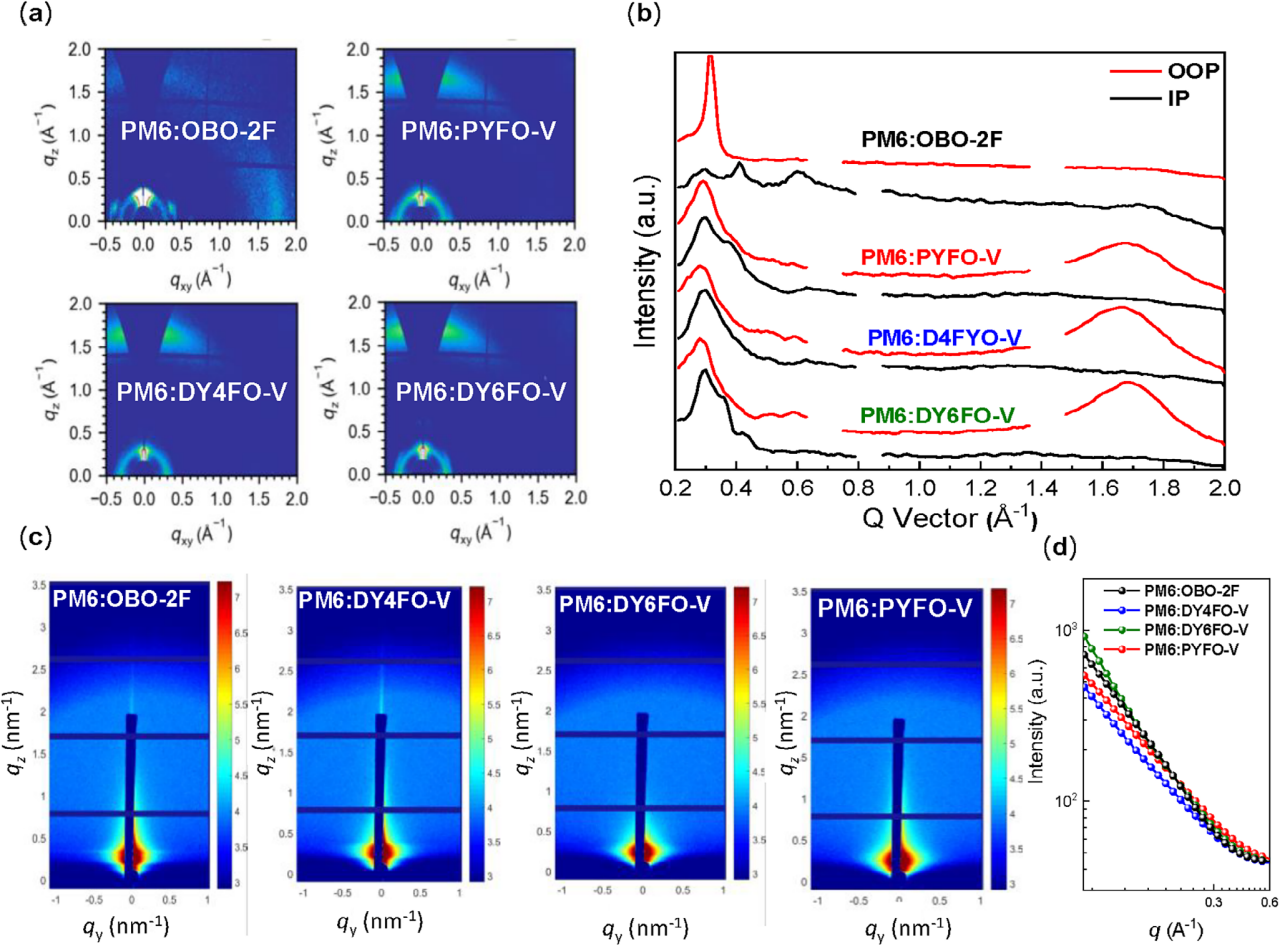
a) 2D GIWAXS patterns of blend films; b) Corresponding line‐cuts in in‐plane (IP) and out‐of‐plane (OOP) directions; c) 2D GISAXS patterns of the blend films and d) the corresponding best fittings along the in‐plane directions.

GIWAXS measurements were performed to further explore the molecular packing differences between these blends. The 2D patterns and line‐cut profiles are displayed in **Figure** **4**a,b, with the corresponding fitting parameters in Table S3 (Supporting Information). As shown in Figure 4a,b, except for the PM6:OBO‐2F, the other three blends demonstrated pronounced and dominant face‐on molecular orientation, which is favorable for charge transportation and collection. In the OOP direction, the *D*‐spacing for DY4FO‐V‐, DY6FO‐V‐, and PYFO‐V‐ based blends are 3.76, 3.70, and 3.72 Å, respectively. In addition, the DY6FO‐V‐based blend film exhibits a larger CCL of 25.7 Å compared to PM6:DY4FO‐V (22.6 Å) and PM6:PYFO‐V(20.9 Å). This demonstrates that the GDA‐based blends possess a more denser packing mode along the π–π direction, resulting in the fastest charge transport as above discussed. Subsequently, the surface morphology were further investigated by atomic force microscopy (AFM). As shown in Figure S21 (Supporting Information), the lowest root‐mean‐square roughness (RMS) of the PM6:DY6FO‐V blend with a favorable nano‐fibrillar network, referring to a better‐kept morphology. While the oversized RMS for the PM6:OBO‐2F derived from the non‐ideal phase separation, is detrimental to the charge extraction and thus a poor PCE achieved in the corresponding devices.

To deeply study the phase separation of these blends, the grazing small‐angle X‐ray scattering (GISAXS) was processed and the obtained 2D‐patterns and IP intensity profiles are shown in Figure 4c,d. To quantify the average domain size of the mixing (*ξ*) and pure phases (2*R*_g_), we fitted the intensity profiles with the Debye–Anderson‐Brumberger (DAB) model and the fractal‐like network model. Detailed calculation results are summarized in Table S7 (Supporting Information). Apparently, the SMA‐based blend possessed oversized scale of the mixing and pure phases, supporting the unfavorable phase separation within blend film found in the AFM image, which should be the major reason for the poor device performance of PM6:OBO‐2F. As the molecular weight increased, both *ξ* and 2*R*_g_ significant reduced to 21–22 nm and 26–27 nm, implying an appropriate phase segregation. According to the GIWAXS, AFM, and GISAXS characterizations, the DY6FO‐V‐based blend possesses a superior crystallinity, desirable phase segregation and smooth surface, ensuring efficient charge transport and suppressed charge recombination.

### 2.5 Indoor Photovoltaic Performance

**Figure 5:**
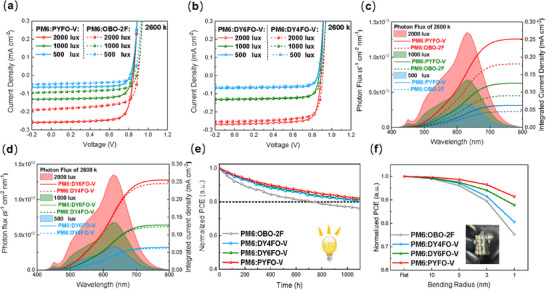
The *J–V* curves of IOPVs based on a) PM6: PYFO‐V/OBO‐2F and b) PM6: GDAs under different indoor light intensity. c) and d) Photon flux of the 2600 k indoor spectrum and integrated current density of PM6:acceptors. e) MPP stability test of the devices under simulated LED light soaking condition. f) Mechanical stabilities of the devices in different radii with bending cycles with 250 bending cycles, respectively.

The indoor performance of these alkoxy acceptors were investigated under 2600 k LED lamps based on different light intensities (2000, 1000, and 500 lux). The input power measurement and indoor PCE calculations followed the procedure in our previous works.^[^ ^4^, ^14^, ^50^ ^]^ **Figure** **5**a,b plotted the *J–V* curves of these alkoxy acceptor‐based devices at different illuminance, and Figure 5c,d depict the corresponding photon flux and integral current density, showing an evident integral current density superiority of DY6FO‐V‐based devices. As detailed IOPV parameters summarized in **Table** **3**, when the 1‐sun illumination is changed to LED lighting conditions, the *V*_OC_ of all devices decreases unavoidably, because the intensities of lighting sources drop significantly.^[^ ^51^ ^]^ PM6:PYFO‐V exhibits PCEs of 26.0% and 23.9% at a luminous intensity of 2000 and 500 lux, respectively. However, under the illumination of 2000 lux, the PM6:OBO‐2F device yielded a non‐ideal efficiency of 17.5%, due to its poor FF and *J*_SC_ which may related to severe recombination and deficient capability of photocurrent generation. Impressively, the DY6FO‐V‐based device achieved a remarkable PCE of 29.1% with a *V*_OC_ of 0.891 V, a *J*_SC_ of 0.267 mA cm^−2^ and an FF of 77.8% under 2000 lux, which is the best PCE among giant molecule acceptors ‐based binary IOPVs (Table S2, Supporting Information). Notably, despite the comparable *V*_OC_ of PM6:DY6FO‐V to the PM6:DY4FO‐V, the much higher *J*_SC_ and FF in PM6:DY6FO‐V lead to a significant increase in IOPV performance. More importantly, the PM6:DY6FO‐V devices can maintain high efficiencies ≈ 27.4–29.1% in a wide range of light intensity (500 to 2000 lux), which could be ascribed to the smaller leakage current (Figure S22, Supporting Information) and less trap‐assisted recombination (Figure 3g), indicating huge potential of DY6FO‐V in IOPVs for IoTs.

**Table 3:** Photovoltaic parameters of the devices under 2600 K indoor spectrum with different light intensities.

Active layer	Light intensity [lux]	*P*_in_ [mW cm^−2^]	*V*_OC_ [V]	*J*_SC_/*J*_cal_ [mA cm^−2^]	FF [%]	PCE [%]
PM6:OBO‐2F	500	0.159	0.846	0.046/0.045	61.4	15.2 (15.0 ± 0.4)
	1000	0.318	0.880	0.092/0.090	66.0	16.9 (16.6 ± 0.3)
	2000	0.637	0.895	0.185/0.181	67.1	17.5 (17.3 ± 0.3)
PM6:DY4FO‐V	500	0.159	0.868	0.062/0.061	71.6	24.2 (23.9 ± 0.3)
	1000	0.318	0.893	0.127/0.122	74.9	26.7 (26.4 ± 0.3)
	2000	0.637	0.912	0.252/0.244	75.6	27.2 (27.0 ± 0.3)
PM6:DY6FO‐V	500	0.159	0.855	0.068/0.063	74.7	27.4 (27.2 ± 0.3)
	1000	0.318	0.872	0.131/0.127	76.4	27.5 (27.3 ± 0.2)
	2000	0.637	0.891	0.267/0.255	77.8	29.1 (28.8 ± 0.2)
PM6:PYFO‐V	500	0.159	0.813	0.064/0.063	72.7	23.9 (23.7 ± 0.3)
	1000	0.318	0.836	0.130/0.126	73.3	25.1 (24.9 ± 0.3)
	2000	0.637	0.849	0.258/0.251	75.5	26.0 (25.7 ± 0.3)

The brackets contain averages and standard errors of PCEs based on 10 devices.

In addition, device stability is another crucial factor for the commercialization of IOPVs, so we compared the long‐term operational stability of well‐encapsulated devices under LED light‐soaking condition (Figure 5e). Detailed stability test methods are provided in the Supporting Information. The PCE of the PM6:GDAs/PA device maintained ≈81% of the original values after 1100 h, higher than the PM6:OBO‐2F (76%), demonstrating the feasibility of practical indoor applications of the DY6FO‐V and DY4FO‐V. The better photostability observed in GDAs/PA‐ based IOPVs could be attributed to the slow diffusion rate, reduced energy disorder and higher *T*_g_ values. In addition, we conducted thermal stability tests on these devices under 85 °C hotplate (Figure S23, Supporting Information). We observed that the efficiency of PM6:GDAs/PA‐based devices maintained ≈ 92% of its initial value after 350 h. In contrast, the OBO‐2F‐based device retained only 83% of its initial PCE after the same time of annealing at 85 °C, due to the low *T*_g_ valve of OBO‐2F. These results demonstrate that enhanced thermal stability of devices can be achieved by the use of acceptors with high *T*_g_s. Subsequently, the flexible devices were fabricated with a structure of polyethylene naphthalate (PEN)/ITO/PEDOT:PSS/active layer/PDINN/Ag to further investigate the mechanical stability in flexible devices. All the flexible devices were mechanically bent with different bending radii (*r*, from 10 to 1 mm, Figure 5f) for 250 circles.^[^ ^52^ ^]^ It's obvious that with the increasement in molecular weight of acceptor materials, the mechanical stability can be significantly enhanced. And PM6:DY6FO‐V based device maintained over 88% of the initial PCE even when *r* is as small as 1 mm, outperforming the DY4FO‐V and OBO‐2F based device. Such a good mechanical performance is mainly attributed to the elasticity of the DY6FO‐V that enables the flexible solar cells to be more robust to mechanical bending. In summary, the stability results suggested that DY6FO‐V is an excellent GDA for future application of the IOPVs with long‐term operation and mechanical stability.

## 3 Conclusion

In this work, in order to simultaneously enhance the efficiency and stability of IOPVs for further IoT applications, we proposed two GDAs (DY4FO‐V and DY6FO‐V) with hypsochromic absorption and high *T*_g_s through a facile synthesis approach. GDAs with definite chemical structures proved to combine the merits of both highly developed small‐molecule and polymeric acceptors. Besides, our new GDAs possessed better crystallinity, thus faster carrier transport and smaller non‐radiative recombination loss than small‐molecule and polymer acceptor‐based devices. Throughout tailoring the fluorination degree, the multi‐fluorinated DY6FO‐V‐based device achieved a decent PCE of 16.6% with promising *V*_OC_ and FF value for further indoor application. As a result, the optimized DY6FO‐V‐based IOPVs realized an impressive PCE of 29.1% under indoor illumination. Meanwhile, the DY6FO‐V‐based devices achieved both better light/thermal stability and mechanical stability compared to small molecule acceptor counterpart. Our study highlights the importance of developing hypsochromic GDAs for highly efficient IOPVs with superior stability.

## Conflict of Interest

The authors declare no conflict of interest.

## Supporting information



Supporting Information

Supporting Information

## Data Availability

The data that support the findings of this study are available in the supplementary material of this article.
