# Supplementary material for: Indoor Organic Photovoltaics with Over 29% Efficiency and Great Stability Enabled by Giant Dimeric Acceptors with Hypsochromic Absorption and High Glass Transition Temperature
Source: Adv Sci (Weinh). 2025 Oct 15;12(44):e12690. doi: 10.1002/advs.202512690 (PMC12667510; doi:10.1002/advs.202512690)
Supplement: Supplementary file 1 — Supporting Information [file ADVS-12-e12690-s001.docx]

Supporting Information:

Indoor Organic Photovoltaics with over 29% Efficiency and Great Stability Enabled by Giant Dimeric Acceptors with Hypsochromic Absorption and High Glass Transition Temperature

Bosen Zou ^†[a,b]^, Ho Ming Ng ^†[b]^, Zhengkai Li ^†[a]^, Yan Wang ^[c]^, Qingyuan Wang ^[a]^, Dezhang Chen ^[d]^, Zefan Yao ^[e]^, Hongxiang Li ^[f]^, Chunliang Li ^[b]^, Xianghao Zeng ^[b]^, Wei Liu ^[b]^, Jonathan E. Halpert ^[d]^, Huawei Hu ^[g]^, Chunhui Duan ^[h]^, Zonglong Zhu ^[c]^, Tom Wu ^[i]^, Wai-Yeung Wong ^[j]^, Zhi-Guo Zhang ^* [a]^, and He Yan ^*[b]^ Han Yu ^* [b,j]^

[a] Dr. B. Zou ^†^, Z. Li ^†^, Q. Wang, Prof. Z.-G. Zhang*

State Key Laboratory of Organic/Inorganic Composites, Beijing Advanced Innovation Center for Soft Matter Science and Engineering

Beijing University of Chemical Technology

Beijing 100029 (China)

Email: zgzhangwhu@iccas.ac.cn

[b] Dr. B. Zou ^†^, H. M. Ng ^†^, Prof. H. Yu*, C. Li, X. Zeng, Dr. W. Liu, Prof. H. Yan*

Department of Chemistry and Hong Kong Branch of Chinese National Engineering Research Center for Tissue Restoration and Reconstruction

The Hong Kong University of Science and Technology

Hong Kong 999077 (China)

E-mail: hyan@ust.hk; yuhan.yu@polyu.edu.hk

[c] Y. Wang, Prof. Z. Zhu

Department of Chemistry and Hong Kong Institute for Clean Energy

City University of Hong Kong

Hong Kong 999077 (China)

[d] Dr. D. Chen, Prof. J. E. Halpert

Department of Chemistry

The Hong Kong University of Science and Technology

Hong Kong 999077 (China)

[e] Dr. Z. Yao

College of Chemistry and Molecular Engineering

Peking University

Beijing 100029 (China)

[f] Dr. H. Li

College of Polymer Science and Engineering, State Key Laboratory of Polymer Materials Engineering

Sichuan University

Chengdu 610106 (China)

[g] Prof. H. Hu

State Key Laboratory for Modification of Chemical Fibers and Polymer Materials, College of Materials Science and Engineering

Donghua University

Shanghai 201620 (China)

[h] Prof. C. Duan

State Key Laboratory of Luminescent Materials and DevicesGuangdong Basic Research Center of Excellence for Energy & Information Polymer Materials

South China University of Technology

Guangzhou 510640 (China)

[i] Prof. Tom. Wu

Department of Applied Physics

The Hong Kong Polytechnic University

Hong Kong 999077 (China)

[j] Prof. H. Yu, Prof. W. Wong

Department of Applied Biology and Chemical Technology and Research Institute for Smart Energy

The Hong Kong Polytechnic University

Hong Kong 999077 (China)

[^†^] These authors contributed equally to this work.

General information. Nuclear magnetic resonance (NMR) spectra were recorded on a Bruker AV-400 MHz NMR spectrometer. Mass spectra were collected on a MALDI MicroMX mass spectrometer, or an API QSTAR XL System.

Material synthesis and Characterizations. All chemicals, unless otherwise specified, were purchased from Energy and used without further purification. The materials (PDINN, IC-FBr, IC-Br, Vinylene-Sn and BTP-OBO-CHO) were purchased from Volt-Amp Optoelectronics Tech. Co., Ltd, Dongguan, China. Toluene and tetrahydrofuran were distilled from sodium benzophenone under nitrogen before using. V-DSEE/FV-DSEE was prepared using the methods reported previously in our team^[1,2]^. PYFO-V and OBO-2F were synthesized as same as previous literatures^[3,4]^. The molecular weight and the PDI of PYFO-V used in this research were 14693 g mol^-2^ and 1.44.

Molecular dynamics simulations and interaction energies estimation. Molecular structures were built and first optimized with deriding force field^[5]^ with Gasteiger charges^[6]^ in Materials Studio package and then subjected to quenching MD simulations at high temperature of 500 K for 1 ns. The generated structures were extracted and sorted by energy to select stable interacted molecules. The molecular structures from MD simulations were further optimized at the level of theory of GFN2-xTB.^[7]^ DFT calculations at B3LYP-D3BJ/def2-SVP were performed in ORCA package to estimate the interaction energies with BSSE correction.^[8]^ Multiwfn and Avogadro were used for input file generation and structure visualization.^[9]^

Thermogravimetric analysis (TGA) measurements. The TGA thermograms of materials were independently tested on the TA Instruments Q5000 SA Thermogravimetric Analyzer in HKUST. The condition is from room temperature to 800 ℃ under Nitrogen atmosphere and the heating rate is 10 ℃ min^-1^. Sapphire and Indium were used to calibrate the baseline and temperature.

Optical characterizations. Film UV-Vis absorption spectra were acquired on a Perkin Elmer Lambda 20 UV/VIS Spectrophotometer. All film samples were spin-cast on ITO substrates. UV-Vis absorption spectra were collected from the solution with the concentration of 1.0 × 10^-5^ M in chloroform. A cuvette with a stopper (Sigma Z600628) was used to avoid volatilization during the measurement. Note : The observed 0-0 and 0-1 absorption peaks in the UV–vis spectra correspond to molecular aggregation types: a higher 0-0/0-1 peak ratio suggests a stronger J-aggregation tendency, featuring as a head-to-tail molecular arrangement with a red-shifted and sharp absorption band, whereas a lower ratio indicates an H-aggregation character, associated with a face-to-face stacking and a blue-shifted absorption band.

Electrochemical characterizations. Cyclic voltammetry was carried out on a CHI610E electrochemical workstation with three electrodes configuration, Ag/AgCl as the reference electrode, Pt plate as the counter electrode, and a glassy carbon as the working electrode. 0.1 mol L^-1^ tetrabutylammonium hexafluorophosphate in anhydrous acetonitrile was used as the supporting electrolyte. The polymer and small molecules were drop-cast onto the glassy carbon electrode from chloroform solutions to form thin films. Potentials were referenced to the ferrocenium/ferrocene couple by using ferrocene as external standards in acetonitrile solutions. The scan rate is 100 mV s^-1^.

Fourier-transform photocurrent spectroscopy external quantum efficiency (FTPS-EQE) and (External quantum efficiency of electroluminescence) EL-EQE measurements. FTPS-EQE was measured using an integrated system (PECT-600, Enlitech), where the photocurrent was amplified and modulated by a lock-in instrument. EL-EQE measurements were performed by applying external voltage/current sources through the devices (REPS-Pro, Enlitech). All of the devices were prepared for EL-EQE measurements according to the optimal device fabrication conditions. EL-EQE measurements were carried out from 0 to 1.8 V.

AFM analysis. AFM measurements were performed by using a Scanning Probe Microscope Dimension 3100 in tapping mode. All film samples were spin-cast on ITO substrates.

Solar cell fabrication and testing. OPVs were made with a device structure of ITO/PEDOT:PSS/ active layer/PDINN/Al, all devices are measured under AM 1.5G, 100 mW cm^−2^ illumination. The glass substrate was coated with indium tin oxide (ITO, 15 Ω square^-1^) (device area: 0.04 cm^2^). The substrate was cleaned in the order of dishwashing liquid, deionized water, acetone (20 min), deionized water and isopropyl alcohol (20 min). A thin PEDOT: PSS (CLEVIOSTM P VP AI 4083, Heraeus, Germany) layer with a thickness of about 25 nm was spin-coat onto the ITO substrates at 4500 rpm for 20 s, and then thermal annealing at 150 ℃ for 15 min in air and the substrate was then transferred to the glove box for active layer deposition. The donor: acceptor blends with weight ratio of 1:1.2 were dissolved in chloroform with donor concentration of 7.0 mg mL^-1^. Subsequently, 0.5% (by volume) 1-chloronaphthalene was added as additive in SMAs series, and 1.0% (by volume) 1-chloronaphthalene was added as additive in Dimer and Polymer Acceptors series and then the solution was stirred at 50 ℃ for 2 hours. The active layer solution was spin coated at 3500 rpm for 30 s. Then the PDINN solution (1.0mg mL^-1^ in MeOH) was spin-coated on the top of the active layer at 2000 rpm for 30 s as the electron transport layer. The substrate was then pumped into a high vacuum at a pressure of 2 × 10^-7^ torr, where the Al layer (100 nm) is thermally evaporated onto the active layer.

The flexible device was fabricated following the same route of rigid device except that the substrate was replaced by PEN (polyethylene naphthalate)/ITO flexible substrate. The current-voltage(*J-V*) characteristics of the all-PSC devices were measured by a Keithley 2400 Source Meter under R.T. in the glove box. The bending test were performed by using a bending cycle ring test instrument (Type: KJD-WQ-0513) produced by Shenzhen Ke Jing Da equipment co., LTD. The bending radius was set from 10 mm to 1 mm for 250 bending circles. The tests were interrupted periodically in order to measure the *J–V* characteristics of the flexible devices.

EQE measurements. EQEs were measured using an Enlitech QE-S EQE system equipped with a standard Silcon diode. Monochromatic light was generated from a Newport 300W lamp source.

SCLC measurements

Hole-mobility measurement

The hole-mobilities were measured using the space charge limited current (SCLC) method^[10,11]^ employing a device architecture of ITO/PEDOT:PSS/active layers/MoO_x_/Ag. The mobilities were obtained by taking current-voltage curves and fitting the results to a space charge limited form, where the SCLC is described by:

$$\boldsymbol{J}\mathbf{=}\frac{\mathbf{9}\boldsymbol{\varepsilon}_{\mathbf{0}}\boldsymbol{\varepsilon}_{\mathbf{r}}\boldsymbol{\mu}{\mathbf{(}\boldsymbol{V}_{\mathbf{appl}}\mathbf{-}\boldsymbol{V}_{\mathbf{bi}}\mathbf{-}\boldsymbol{V}_{\mathbf{s}}\mathbf{)}}^{\mathbf{2}}}{\mathbf{8}\boldsymbol{L}^{\mathbf{3}}}$$

Where *ε*_0_ is the permittivity of free space, *ε*_r_ is the relative permittivity of the material (assumed to be 3), *μ* is the hole mobility and *L* is the thickness of the film. From the plots of *J^1/2^* vs $\boldsymbol{V}_{\mathbf{appl}}\boldsymbol{-}\boldsymbol{V}_{\mathbf{bi}}\boldsymbol{-}\boldsymbol{V}_{\mathbf{s}}$, hole mobilities can be deduced.

Electron-mobility measurement

The electron mobilities were measured using the SCLC method, employing a device architecture of ITO/ZnO/active layer/ PNDI-F3N /Ag. The mobilities were obtained by taking current-voltage curves and fitting the results to a space charge limited form, where the SCLC is described by:

$$\boldsymbol{J}\mathbf{=}\frac{\mathbf{9}\boldsymbol{\varepsilon}_{\mathbf{0}}\boldsymbol{\varepsilon}_{\mathbf{r}}\boldsymbol{\mu}{\mathbf{(}\boldsymbol{V}_{\mathbf{appl}}\mathbf{-}\boldsymbol{V}_{\mathbf{bi}}\mathbf{-}\boldsymbol{V}_{\mathbf{s}}\mathbf{)}}^{\mathbf{2}}}{\mathbf{8}\boldsymbol{L}^{\mathbf{3}}}$$

Where *ε*_0_ is the permittivity of free space, *ε*_r_ is the relative permittivity of the material (assumed to be 3), *μ* is the hole mobility and *L* is the thickness of the film. From the plots of *J^1/2^* vs $\boldsymbol{V}_{\mathbf{appl}}\boldsymbol{-}\boldsymbol{V}_{\mathbf{bi}}\boldsymbol{-}\boldsymbol{V}_{\mathbf{s}}$, electron mobilities can be deduced.

Transient photovoltage (TPV) and Transient photocurrent (TPC)

Transient photocurrent (TPC) and transient photovoltage (TPV) measurements were carried out using a 488 nm solid-state laser (Coherent OBIS CORE 488LS) with a pulse width of approximately 30 ns. The current signals were captured by a mixed-domain oscilloscope (Tektronix MDO3032) after converting the voltage drop across a 2 Ω resistor load connected in series with the solar cell. For TPV measurements, the solar cell was maintained under open-circuit conditions using the same pulsed laser, and the photovoltage signals were recorded by the oscilloscope with an external 10 MΩ resistor in series.

GIWAXS and GISAXS measurements and fittings. GIWAXS data were obtained at beamline BL02U2 of Shanghai Synchrotron Radiation Facility (SSRF). The monochromatic of the light source was 1.24 Å. The data were recorded by using the two-dimensional image plate detector of Pilatus 2M from Dectris, Switzerland. GISAXS data of films were obtained at beamline BL16B1 of Shanghai Synchrotron Radiation Facility (SSRF). The monochromatic of the light source was 1.24 Å. The incidence angle was 0.2°, and the sample-to-detector distance was 2200 mm by calibration for GISAXS.

The GISAXS 1D profiles were fitted with a universal model^[12]^ following Equation (1). Data fitting was done using SasView (version 5.01) software.

$$\mathbf{I}\left( \boldsymbol{q} \right)\mathbf{=}\frac{\boldsymbol{A}_{\mathbf{1}}}{\mathbf{[}{\mathbf{1+}{\mathbf{(}\boldsymbol{q\xi}\mathbf{)}}^{\mathbf{2}}\mathbf{]}}^{\mathbf{2}}}\mathbf{+}\boldsymbol{A}_{\mathbf{2}}\boldsymbol{\langle}\mathbf{P}\left( \mathbf{q,R} \right)\boldsymbol{\rangle}\mathbf{S}\left( \boldsymbol{q, R, \eta, D} \right)\mathbf{+B}$$

$$\boldsymbol{S}\left( \boldsymbol{q} \right)\mathbf{=1+}\frac{\mathbf{sin[(}\boldsymbol{D}\mathbf{-1)}\mathbf{tan}^{\mathbf{-1}}\mathbf{(}\boldsymbol{q\eta}\mathbf{)]}\boldsymbol{b}\boldsymbol{\pm}\sqrt{\boldsymbol{b}^{\mathbf{2}}\mathbf{-4}\boldsymbol{ac}}}{{\mathbf{(}\boldsymbol{qR}\mathbf{)}}^{\boldsymbol{D}}} \frac{\boldsymbol{D}\mathbf{Г(}\boldsymbol{D}\mathbf{-1)}}{{\mathbf{[1+}\frac{\mathbf{1}}{{\mathbf{(}\boldsymbol{q\eta}\mathbf{)}}^{\mathbf{2}}}\mathbf{]}}^{{\mathbf{(}\boldsymbol{D}\mathbf{-1)}}/\mathbf{2}}}$$

where A_1_, A_2_, and B are independent fitting parameters and q is the scattering wave vector. The average correlation length ξ of the PM6 domain and the Debye-Andersone-Brumberger (DAB) term make up the first term. The contribution from acceptor fractal-like aggregations is seen by the second term. Here, R is the mean spherical radius of the primary acceptor particles, P (q, R) is the form factor of the acceptor, S (q, R, η, D) is the fractal structure factor to explain the primary particles interaction in this fractal-like aggregation system, η is the correlation length of the fractal-like structure, and D is the fractal dimension of the network. Equation 3 was used to calculate the average domain size by the Guinier radius of the fractallike network *R*_g_.

$$\boldsymbol{R}_{\boldsymbol{g}}\boldsymbol{=\eta}\sqrt{\frac{\boldsymbol{D(D+1)}}{\boldsymbol{2}}}$$

Indoor photovoltaic characterization. The characterization of indoor photovoltaic performance follows the methods outlined in our previous work^[3,13,14]^. To ensure accurate evaluation under indoor lighting conditions, anti-reflective-treated masks and blackened testing boxes are employed, effectively minimizing light scattering and reflections. The integrated current density (*J*_cal_) was obtained based on equations below:

*J*_SC_ (calculated) = $\int\boldsymbol{EQE}\mathbf{(}\boldsymbol{\lambda}\mathbf{)}\mathbf{*}\boldsymbol{\Phi}\left( \boldsymbol{\lambda} \right)\mathbf{*}\boldsymbol{q}$

$$\boldsymbol{H}\mathbf{(}\boldsymbol{\lambda}\mathbf{) =}\boldsymbol{\Phi}\mathbf{(}\boldsymbol{\lambda}\mathbf{)*}\boldsymbol{q}\mathbf{*}\boldsymbol{E}\mathbf{(}\boldsymbol{eV}\mathbf{)}$$

where *Φ*(*λ*), *Η*(*λ*) are the photon flux and the power density of the indoor light sources.

Stability measurement of the devices. The devices for stability measurement were fabricated with an inverted structure of ITO/ZnO/PM6: acceptors/MoO_x_/Ag for preventing diffusion of organic cathode interlayer. The devices were fabricated in a glovebox following the procedure described above. After device preparation, an appropriate amount of photocurable resin was evenly applied onto the silver electrode. A glass coverslip of suitable size was then placed on top, and the resin was fully cured by exposing it to 365 nm ultraviolet (UV) light for 15 minutes. Then, remove the encapsulated devices from the glovebox, and test their light/thermal stability in the air. A multi channel solar cell performance decay test system (PVLT-6001M- 32A, Suzhou D&R Instruments Co. Ltd.) and continuous white LED light with 1000 lux were applied.

Synthesis

Scheme. S1 Synthesis routes of the SMA, GDAs and the PA, where R is 2-octyledecyl chain.

Synthesis of OBO-2F

To a mixture of BTP-OBO-CHO (100 mg, 0.07 mmol) and 2-(5,6-difluoro-3-oxo-2,3-dihydro-1H-inden-1-ylidene)malononitrile (IC-2F 33 mg, 0.14 mmol) in anhydrous CHCl_3_ (10 mL) being heated to 65 ^o^C was added pyridine (1.5 mL) under nitrogen. The mixture was refluxed for 4 hours and then cooled to room temperature, the mixture was poured into CH_3_OH (40 mL) and filtered. After removing the solvent, the residue was purified using column chromatography on silica gel (*n*-hexane: DCM= 2:1, v/v), yielding a blue solid (85 mg, 66%). ^1^H NMR (400 MHz, CDCl_3_) *δ* 9.31 (s, 2H), 8.52 (dd, *J* = 10.0, 6.5 Hz, 2H), 7.64 (t, *J* = 7.6 Hz, 2H), 4.72 (dd, *J* = 11.5, 6.9 Hz, 8H), 2.09 (dd, *J* = 12.2, 6.1 Hz, 4H), 1.46 – 0.68 (m, 120H). MALDI-TOF MS: calculated for: C_108_H_138_F_4_N_8_O_4_S_5_ (M^+^), 1848.6; found, 1848.9.

Synthesis of DY4FO-V, DY6FO-V

To a mixture of BTP-OBO-CHO (1 g, 0.7 mmol) and 2-(5,6-difluoro-3-oxo-2,3-dihydro-1H-inden-1-ylidene)malononitrile (IC-2F 138 mg, 0.6 mmol) in anhydrous toluene (100 mL). Subsequently, BF_3_ ∙ OEt_2_ (7.5mL) and acetic anhydride (7.5 mL) were added, and the reaction mixture was stirred at 0 ^o^C for 15 min and then stirred at room temperature for 25 min. Then, the reaction mixture was dropped into methanol, and the precipitate was collected as a crude product. The residue was purified using column chromatography on silica gel (*n*-hexane: DCM= 2:1, v/v), yielding a dark green solid BTP-OBO-2F-CHO (745 mg, 65%).

BTP-OBO-2F-CHO (745 mg, 0.454 mmol), V-DSEE (166 mg, 0.216 mmol), were dissolved in absolute toluene (35 mL), and BF_3_·C_2_H_5_OC_2_H_5_ (0.08 ml), Ac_2_O (0.3 ml) were added. The mixture was reacted 1 hour at 60 ℃. The reaction mixture was then cooled to room temperature and then centrifugal separation with methanol. The crude product was purified by flash column chromatography with petroleum ether and dichloromethane (1:2) as the eluents to afford the final product DY4FO-V as black solid (744 mg, 90%).

BTP-OBO-2F-CHO (200 mg, 0.122 mmol), FV-DSEE (45.8 mg, 0.058 mmol), were dissolved in absolute toluene (10 mL), and BF_3_·C_2_H_5_OC_2_H_5_ (0.02 mL), Ac_2_O (0.08 mL) were added. The mixture was reacted 1 hour at 60 ℃. The reaction mixture was then cooled to room temperature and then centrifugal separation with methanol. The crude product was purified by flash column chromatography with petroleum ether and dichloromethane (1:2) as the eluents to afford the final product DY4FO-V as black solid (160 mg, 75%).

DY4FO-V: ^1^H NMR (400 MHz, CDCl_3_) *δ* 9.37 (s, 1H), 9.25 (s, 1H), 8.74 (d, *J* = 8.2 Hz, 1H), 8.52 (dd, *J* = 10.0, 6.5 Hz, 1H), 8.07 (s, 1H), 7.88 (d, *J* = 8.3 Hz, 1H), 7.54 (dd, *J* = 14.2, 6.7 Hz, 1H), 7.47 (s, 1H), 4.76 (d, *J* = 6.4 Hz, 4H), 4.72 (d, *J* = 5.8 Hz, 4H), 2.18 – 2.03 (m, 4H), 1.70 – 0.69 (m, 120H). MALDI-TOF MS: calculated for: C_218_H_280_F_4_N_16_O_8_S_10_ (M^+^), 3649.3; found, 3648.9.

DY6FO-V: ^1^H NMR (400 MHz, CDCl_3_) *δ* 9.35 (s, 1H), 9.31 (s, 1H), 8.57 (d, *J* = 8.3 Hz, 1H), 8.53 (dd, *J* = 10.0, 6.5 Hz, 1H), 8.03 (t, *J* = 7.5 Hz, 1H), 7.63 (s, 1H), 7.61 (d, *J* = 7.5 Hz, 1H), 4.75 (d, *J* = 7.3 Hz, 4H), 4.72 (d, *J* = 5.5 Hz, 4H), 2.10 (d, *J* = 5.9 Hz, 4H), 1.45 – 0.74 (m, 120H). MALDI-TOF MS: calculated for: C_218_H_278_F_6_N_16_O_8_S_10_ (M^+^), 3685.3; found, 3684.5.


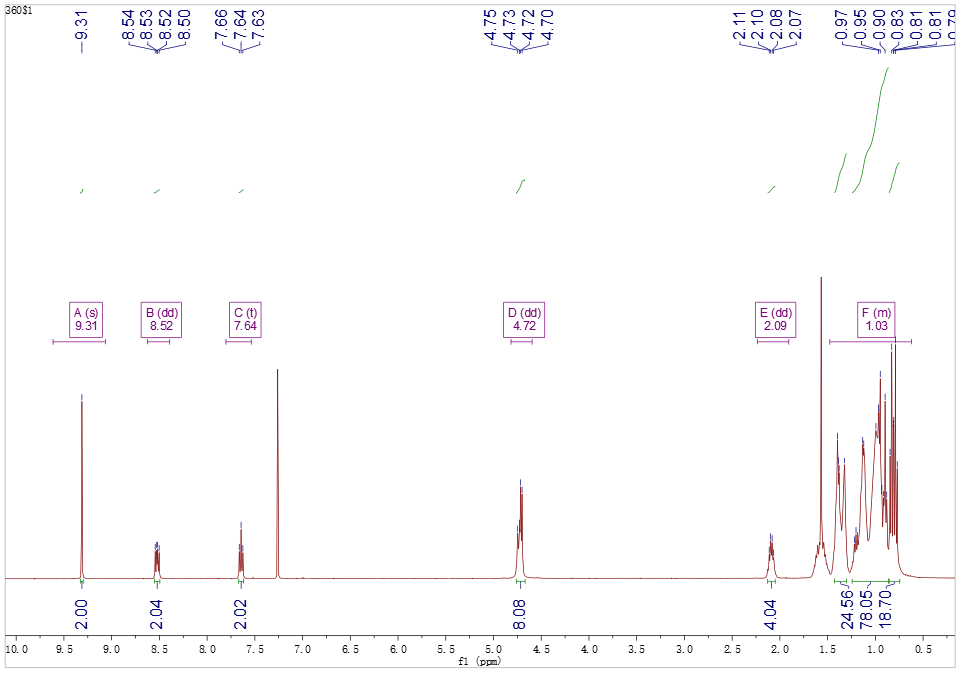


Figure. S1 ^1^H NMR spectrum of OBO-2F (400 MHz, CDCl_3_).


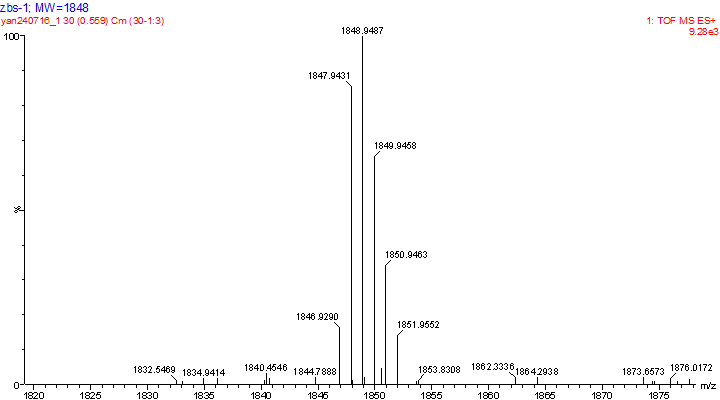


Figure. S2 MS spectrum of OBO-2F.


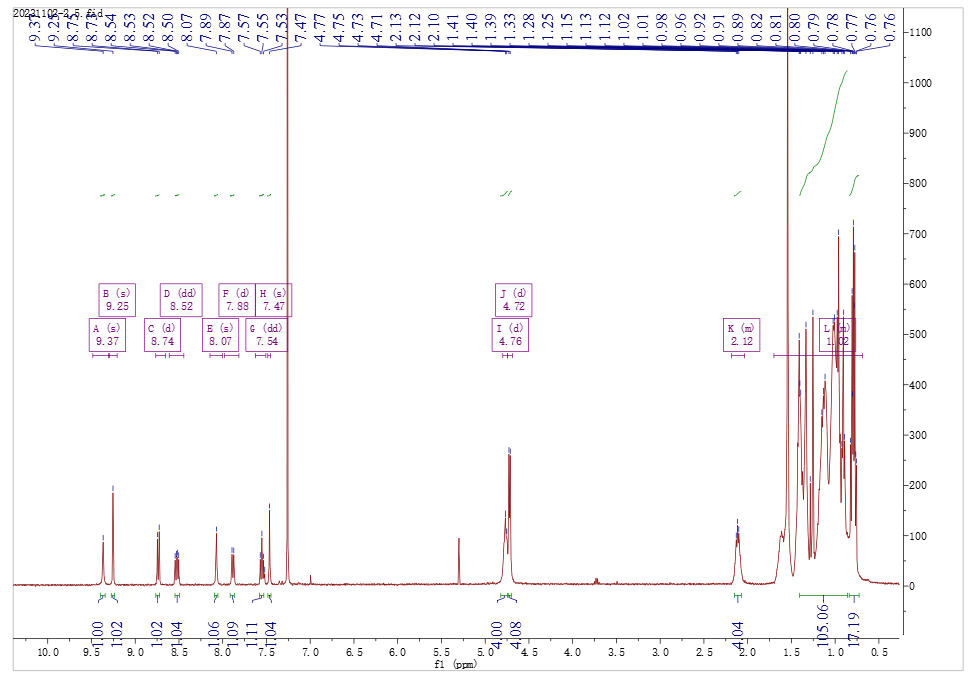


CH_2_Cl_2_

Figure. S3 ^1^H NMR spectrum of DY4FO-V (400 MHz, CDCl_3_).


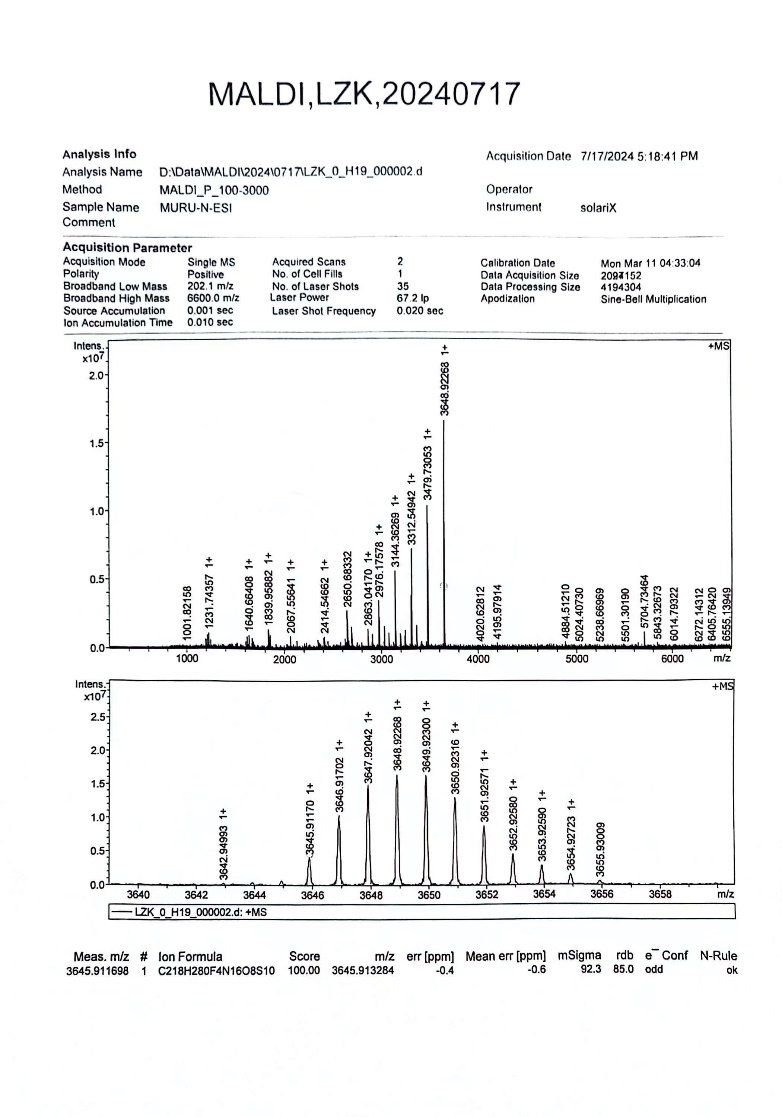


Figure. S4 MS spectrum of DY4FO-V.


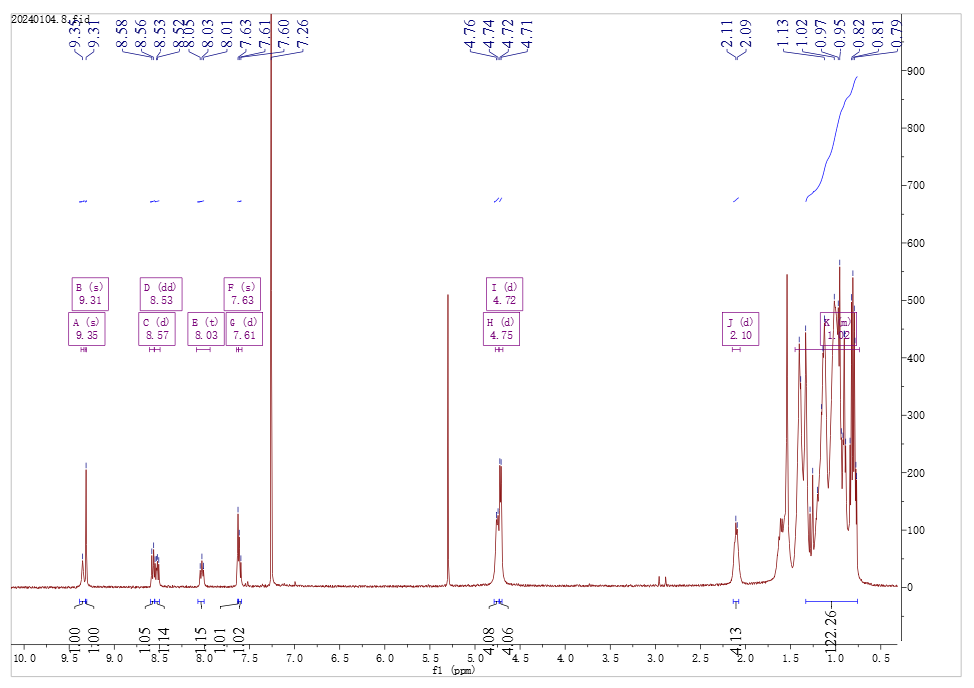


CH_2_Cl_2_

Figure. S5 ^1^H NMR spectrum of DY6FO-V (400 MHz, CDCl_3_).


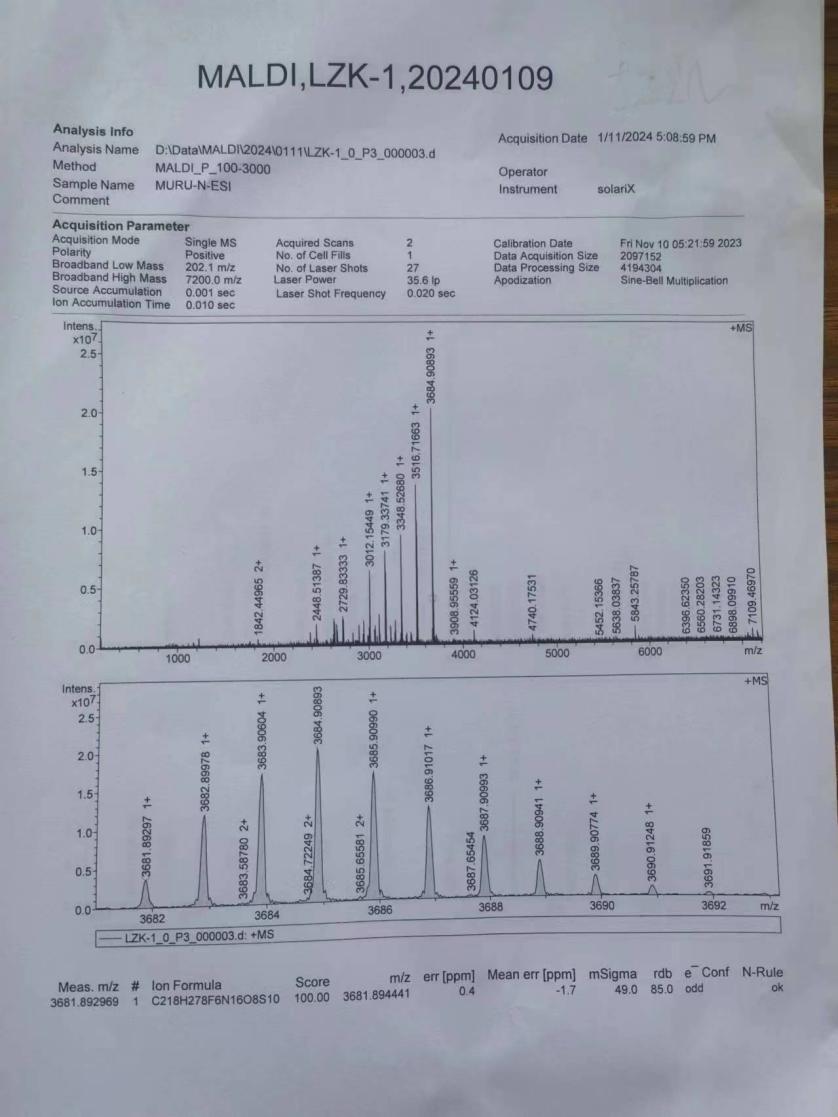


Figure. S6 MS spectrum of DY6FO-V.

Figure. S7 Thermogravimetric analysis (TGA) curves of OBO-2F, DY4FO-V, DY6FO-V and PYFO-V.


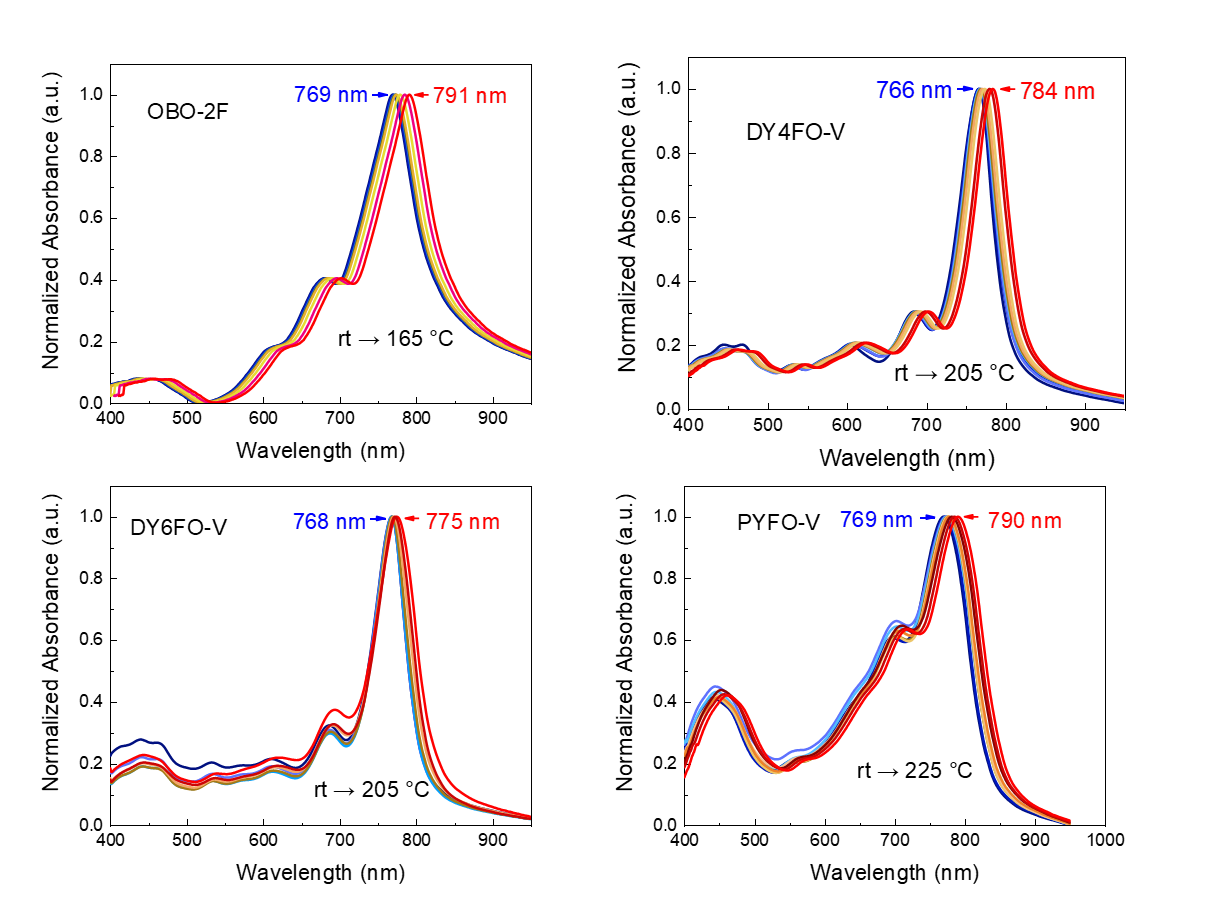


Figure. S8 Temperature varied thermally annealed film’s absorption spectra for *T*_g_ measurement.


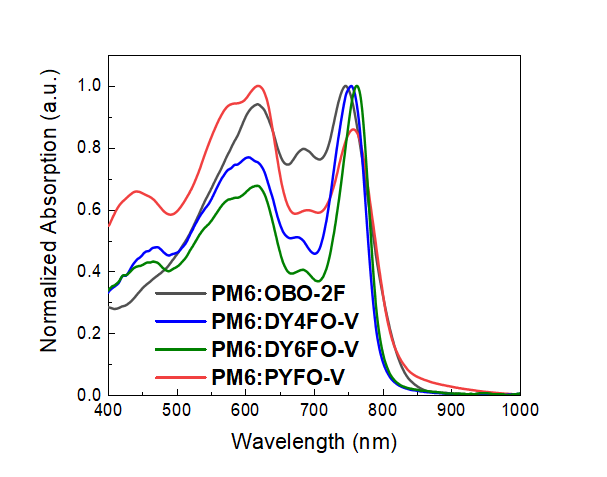


Figure. S9 Normalized UV-Vis absorption spectra of the blends.


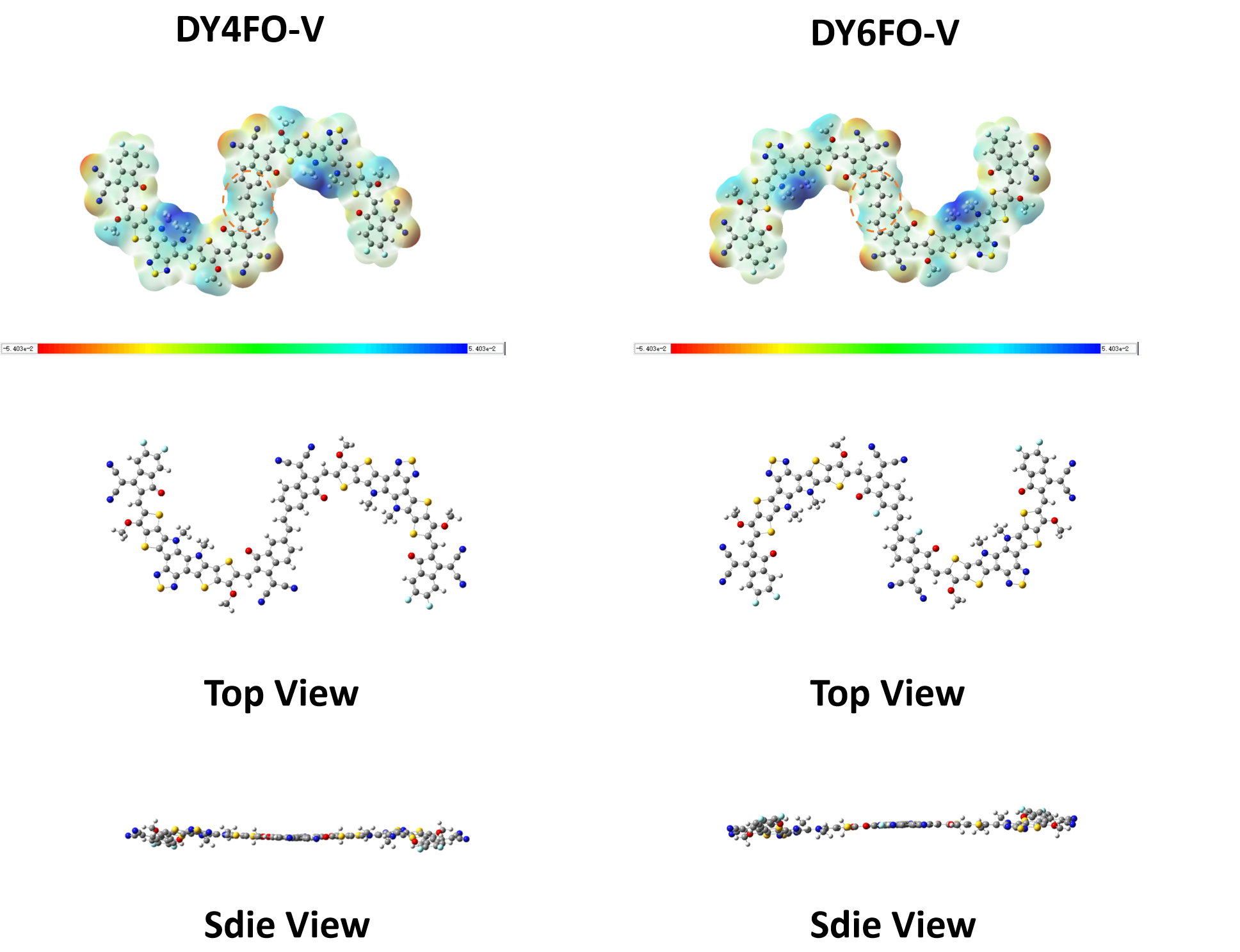


Figure. S10 ESP distributions of DY4FO-V and DY6FO-V calculated by a DFT method at the B3LYP/6-31G(d,p) set.

Figure. S11 Cyclic voltammetry curves of OBO-2F, DY4FO-V, DY6FO-V and PYFO-V.

Figure. S12 Chemical structure of PM6.


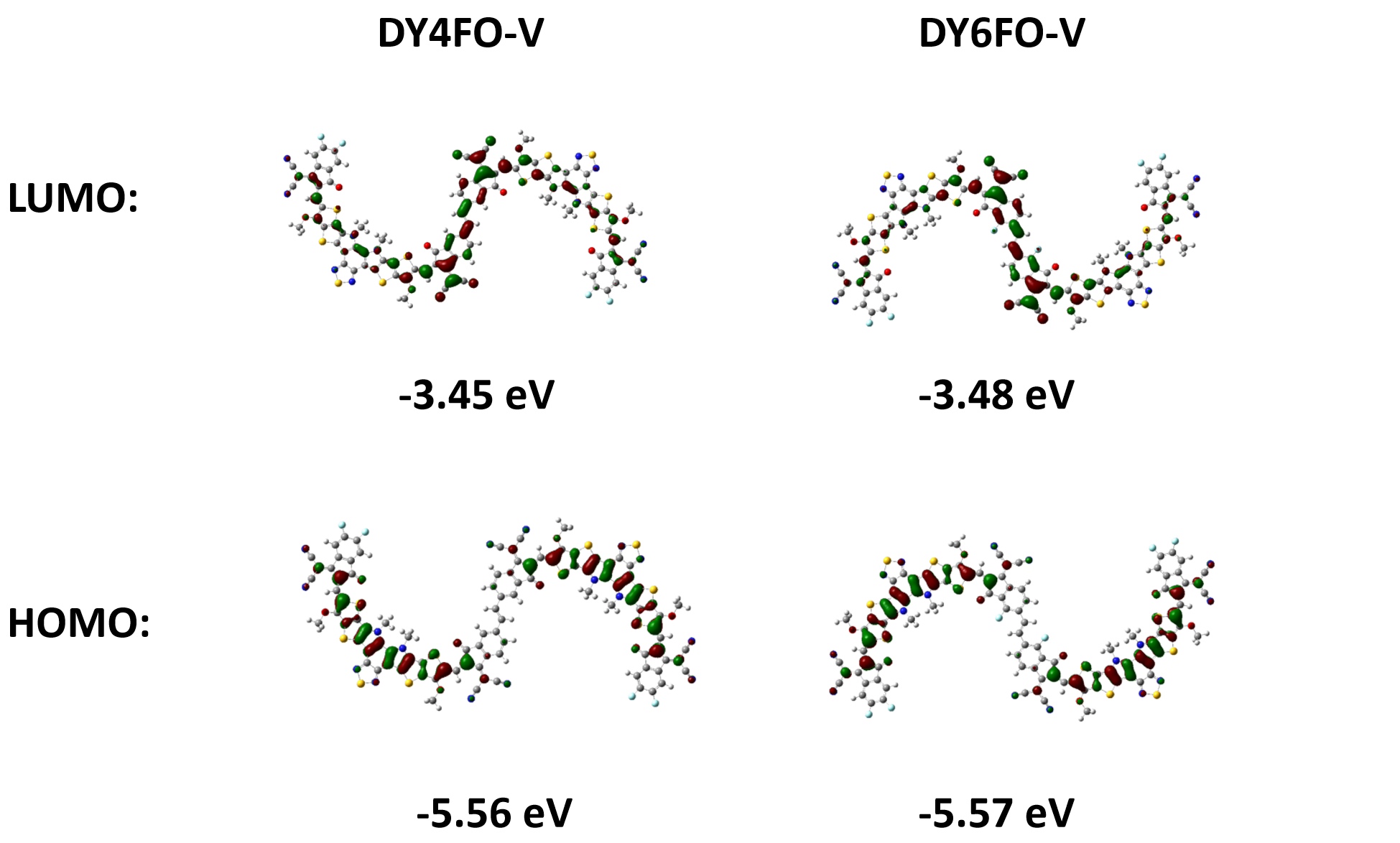


Figure. S13 The calculated frontier orbitals of DY4FO-V and DY6FO-V through the DFT method.

Figure. S14 2D GIWAXS patterns of the pure alkoxy acceptors.

Figure. S15 1D GIWAXS profiles of the pure alkoxy acceptors.


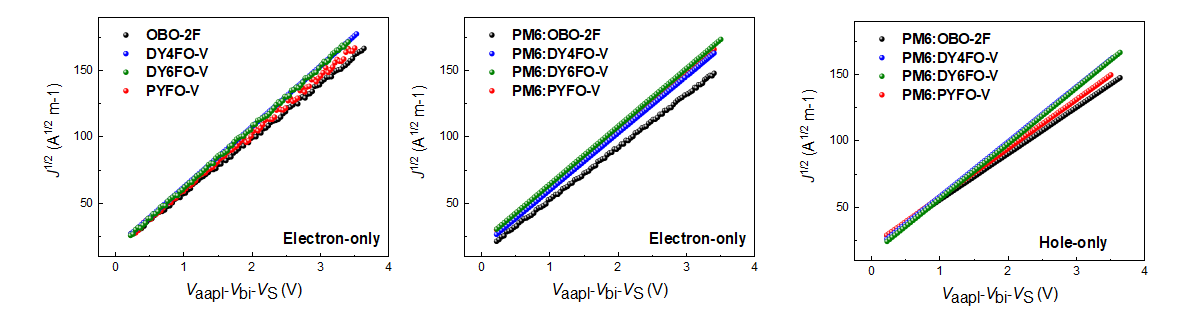


Figure. S16 Hole-only and electron-only device results through SCLC method.

Figure. S17 Normalized electroluminescence (EL) and external quantum efficiency (EQE) spectra of PM6:NFAs based devices for accurate bandgap calculation.


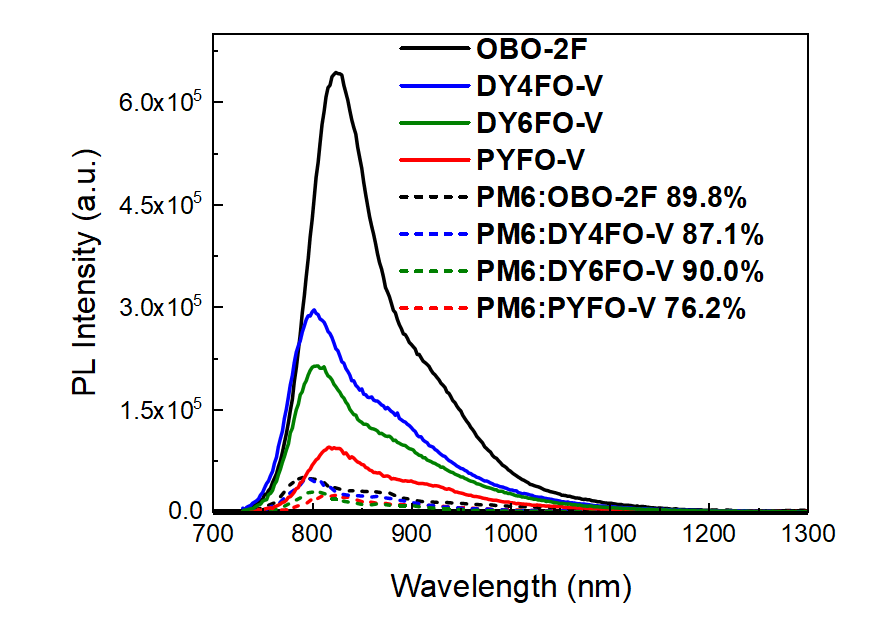


Figure. S18 Photoluminescence spectra of the pure and blend films excited at 785 nm.

Figure. S19 (a) *J*_SC_ as a function of light intensity and power-law fit (*J*_SC_∝ intensity^α^) for different devices and (b) *V*_OC_ as a function light intensity and the logarithmic fit used to determine the ideality factor.

Figure. S20 Film Formation Kinetics. (a) The color mapping of *in-situ* UV–Vis absorption spectra as a function of spin-coating time for blends; (b) Temporal evolution of peak positions of acceptors in blend films.

Figure. S21 AFM height images of all blended films.

Figure. S22 The dark current density for the corresponding devices.


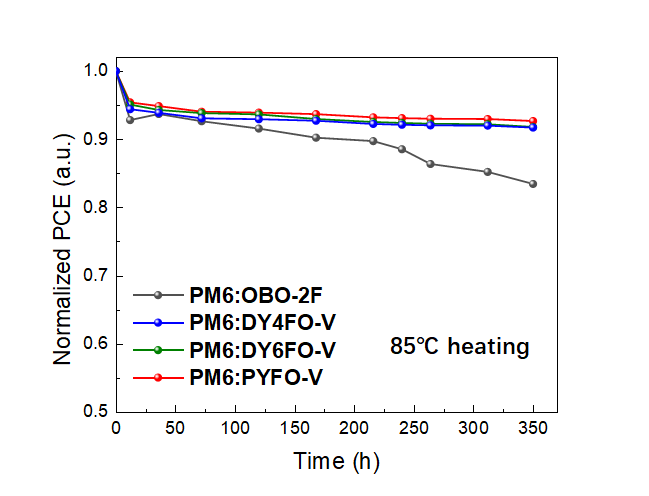


Figure. S23 Normalized PCE plotted against aging time under 85 °C heating condition.

Table. S1 The summarized photovoltaic data of the reported Y-series dimeric acceptor-based binary devices.

| Activelayer | *V*_OC_ (V) | *J*_SC_ (mA cm^-2^) | | | FF (%) | PCE (%) | Ref |
| --- | --- | --- | --- | --- | --- | --- | --- |
| PM6:DYF-V | 0.89 | | 26.6 | 78.6 | | 18.6 | ^[15]^ |
| PM6:DY2F-V | 0.84 | | 26.8 | 76.2 | | 17.2 | ^[15]^ |
| D18:DY-FT | 0.93 | | 23.8 | 68.6 | | 15.3 | ^[16]^ |
| D18:DY-IDT | 0.98 | | 19.1 | 64.1 | | 12.0 | ^[16]^ |
| PPM6:DQx-Ph | 0.962 | | 20.1 | 67.4 | | 13.0 | ^[17]^ |
| MPhS-C2:Se-Giant | 0.918 | | 25.2 | 70.3 | | 16.3 | ^[18]^ |
| PM6:GMA-SSS | 0.913 | | 27.13 | 75.35 | | 18.66 | ^[19]^ |
| PM6:GMA-SSeS | 0.917 | | 27.38 | 19.37 | | 19.37 | ^[19]^ |
| PM6:GMA-SeSSe | 0.882 | | 27.47 | 18.17 | | 18.17 | ^[19]^ |
| PM6:GMA-SeSeSe | 0.865 | | 26.91 | 16.49 | | 16.49 | ^[19]^ |
| PM6:T0 | 0.923 | | 24.1 | 77.1 | | 17.1 | ^[20]^ |
| PM6:T1 | 0.959 | | 21.2 | 71.7 | | 14.6 | ^[20]^ |
| PM6:T4 | 0.962 | | 22.5 | 76.6 | | 16.6 | ^[20]^ |
| PM6:T6 | 0.970 | | 22.9 | 76.8 | | 17.1 | ^[20]^ |
| PM6:T12 | 0.980 | | 21.3 | 70.9 | | 14.8 | ^[20]^ |
| PM6:D-TPh | 0.946 | | 25.6 | 78.7 | | 19.1 | ^[21]^ |
| PM6:D-TN | 0.930 | | 25.3 | 78.2 | | 18.4 | ^[21]^ |
| PM6:DYV | 0.910 | | 25.97 | 76.22 | | 18.01 | ^[22]^ |
| PM6:DYVC | 0.915 | | 24.87 | 73.86 | | 16.81 | ^[22]^ |
| PM6:DYTVT  PM6:DYC10  D18:DY-T  D18:DY-TF  D18:DYF-TF  PM6:DIBP3F-Se  PM6:DIBP3F-S  PM6:DYBO  PM6:Dimer-QX  PM6:Dimer-2CF  D18:DYA-I  D18:DYA-IO  D18:DYA-O  PM6:BT-DL  PM6:B-DL  PM6:dBTICy-EH  PM6:dBTICy-BO  PM6:2BTP-2F-T  PM6:RCM  D18/2Y-end  D18:DBY-2Cl  D18:DTY-2Cl  PM6:QM1  PM6:QM2  PBQx-H-TF: dBTIC-𝛿V-BO  PBQx-H-TF: dBTIC-𝛾V-BO  PBQx-H-TF: dBTIC-𝛾V-OD-2Cl  PBDB-T: OY2  PM6:EV-i  PM6:EV-o  PM6: DYSe-I | 0.935  0.947  0.949  0.945  0.939  0.917  0.901  0.968  0.933  0.889  0.938  0.948  0.961  0.940  0.949  0.92  0.91  0.911  0.979  0.850  0.927  0.913  0.91  0.91  0.96  0.91  0.87  0.837  0.897  0.957  0.94 | | 22.90  22.28  22.57  24.36  25.82  25.92  24.37  24.62  22.57  25.27  25.04  24.10  23.09  25.52  18.56  21.43  20.97  25.50  22.7  27.66  20.76  26.20  25.23  25.42  20.67  24.52  24.65  24.37  26.60  6.20  23.5 | 68.15  68.66  72.47  72.87  75.30  76.1  72.0  75.8  69.26  80.62  0.78  0.76  0.73  77.08  52.11  73.28  70.26  78.28  63.8  0.754  68.76  75.46  74.01  70.50  66.06  76.58  74.51  71.79  16.56  42.13  0.74 | | 14.59  14.48  15.52  16.77  18.26  18.09  16.11  18.08  14.59  18.12  18.83  17.54  16.45  18.49  9.17  14.48  13.42  18.19  14.2  17.73  13.23  18.06  17.05  16.36  13.15  17.14  16.04  14.69  18.27  2.50  16.8 | ^[22]^  ^[22]^  ^[23]^  ^[23]^  ^[23]^  ^[24]^  ^[24]^  ^[25]^  ^[26]^  ^[26]^  ^[27]^  ^[27]^  ^[27]^  ^[1]^  ^[1]^  ^[28]^  ^[28]^  ^[29]^  ^[30]^  ^[31]^  ^[32]^  ^[32]^  ^[33]^  ^[33]^  ^[34]^  ^[34]^  ^[34]^  ^[35]^  ^[36]^  ^[36]^  ^[37]^ |
| PM6:DY4FO-V | 1.00 | | 20.1 | 73.0 | | 14.5 | This |
| PM6:DY6FO-V | 0.99 | | 22.1 | 75.5 | | 16.6 | Work |

Table. S2 The summarized photovoltaic data of the reported high-performance binary IOPVs.

| Active layer | Light intensity (lux) | *V*_OC_ | *J*_SC_ | FF | PCE | Stability | Ref |
| --- | --- | --- | --- | --- | --- | --- | --- |
|  |  | (V) | (mA cm^-2^ ) | (%) | (%) |  |  |
| *Small molecule acceptor-based binary IOPVs* |  |  |  |  |  |  |  |
| PBDB-TF:Y6 | 500 | 0.7 | 0.063 | 74.8 | 20.9 | *T*_80_≈1000 hr | ^[38]^ |
| PB2:FCC-Cl | 500 | 0.909 | 0.064 | 78.9 | 29 | T_82_≈1000 hr | ^[38]^ |
| PBDB-TF:Y6 | 20,000 | 0.812 | 2.54 | 78.4 | 25.6 | *T*_82_≈1000 hr | ^[38]^ |
| PB2:FCC-Cl | 20,000 | 1.02 | 2.53 | 80.5 | 33 | *T*_84_≈1000 hr | ^[38]^ |
| PM6:IT-4F | 1000 | 0.71 | 0.14 | 68.7 | 23.75 | *T*_92_≈600 hr | ^[39]^ |
| D18:GWQ20 | 2000 | 0.864 | 0.248 | 79.8 | 26.8 | / | ^[40]^ |
| D18:Z3 | 2000 | 0.88 | 0.123 | 71.9 | 12.2 | / | ^[40]^ |
| D18:Z4 | 2000 | 0.882 | 0.207 | 76.4 | 21.9 | / | ^[40]^ |
| D18:Z5 | 2000 | 0.885 | 0.194 | 75 | 20.2 | / | ^[40]^ |
| D18:FCC-Cl | 2000 | 0.975 | 0.245 | 80.1 | 30.1 | *T*_95_≈500 hr | ^[41]^ |
| PM6:FCC-Cl | 2000 | 0.914 | 0.244 | 81.2 | 28.5 | / | ^[41]^ |
| D18:FCC-Cl-4Ph | 2000 | 0.995 | 0.218 | 79.6 | 29.5 | *T*_90_≈1000 hr | ^[13]^ |
| D18:FCC-Cl-6Ph | 2000 | 1.02 | 0.189 | 70 | 22.9 | / | ^[13]^ |
| PB4:FTCC-Br | 1000 | 0.967 | 0.119 | 82 | 31.1 | / | ^[42]^ |
| PBDB-TF:IO-4Cl | 1000 | 1.1 | 0.091 | 79.1 | 26.1 | *T*_98_≈1000 hr | ^[43]^ |
| PM6: TB-S | 1000 | 0.832 | 0.116 | 74.1 | 23.3 | / | ^[44]^ |
| *Giant molecule acceptors -based binary IOPVs* |  |  |  |  |  |  |  |
| P3TEA: FTTBPDI4 | 1650 | 1.02 | 0.196 | 67 | 26.67 | / | ^[45]^ |
| P3TEA: FTTBPDI4 | 1200 | 1.02 | 0.143 | 67 | 26.18 | / | ^[45]^ |
| P3TEA: FTTBPDI4 | 700 | 0.99 | 0.079 | 67 | 24.71 | / | ^[45]^ |
| PPDT2FBT: tPDI_2_N-EH | 1000 | 0.88 | 0.187 | 50.4 | 9 | / | ^[46]^ |
| PPDT2FBT: tPDI_2_N-EH | 3000 | 0.82 | 0.04 | 50.5 | 9.2 | / | ^[46]^ |
| PM6:DY4FO-V | 2000 | 0.912 | 0.252 | 75.6 | 27.2 | Operation Stability:  *T*_81_≈1100 hr | This |
| PM6:DY6FO-V | 2000 | 0.891 | 0.267 | 77.8 | 29.1 | Thermal Stability:  *T*_92_≈350 hr | Work |

Table. S3 Calculated parameters for (010) peak from OOP direction.

| Sample | Peak position (Å^-1^) | | *D*-spacing (Å) | CL (Å) |  |
| --- | --- | --- | --- | --- | --- |
| OBO-2F | | / | / | / |  |
| DY4FO-V | | 1.67 | 3.76 | 28.3 |  |
| DY6FO-V | | | 1.69 | 3.72 | 33.3 |
| PYFO-V  PM6:OBO-2F | | 1.68  / | 3.74  / | 21.7  / |  |
| PM6:DY4FO-V | | 1.67 | 3.76 | 22.6 |  |
| PM6:DY6FO-V  PM6:PYFO-V | | 1.70  1.69 | 3.70  3.72 | 25.7  20.9 |  |

Table. S4 Mobilities.

| Active layer | *μ*_e_ (10^-4^ cm^2^ V^-1^ s^-1^) | *μ*_h_ (10^-4^ cm^2^ V^-1^ s^-1^) | *μ*_e_/*μ*_h_ |
| --- | --- | --- | --- |
| OBO-2F | 5.8 | W/O | / |
| DY4FO-V | 6.9 | W/O | / |
| DY6FO-V | 7.0 | W/O | / |
| PYFO-V | 6.5 | W/O | / |
| PM6:OBO-2F | 5.2 | 3.9 | 1.33 |
| PM6:DY4FO-V | 6.2 | 5.6 | 1.11 |
| PM6:DY6FO-V  PM6:PYFO-V | 6.3  5.7 | 5.8  4.7 | 1.09  1.21 |

Table. S5 he overall *E*_loss_ and different contributions to *E*_loss_ in the OSCs.

| Active layer | *E*_g_ (eV) | | *qV*_OC_ (eV) | *E*_loss_ (eV) | *ΔE*_1_ (eV) | *ΔE*_2_ (eV) | *ΔE*_3_ (eV) |
| --- | --- | --- | --- | --- | --- | --- | --- |
| PM6:OBO-2F | 1.555 | 0.973 | | 0.582 | 0.270 | 0.058 | 0.254 |
| PM6:DY4FO-V  PM6:DY6FO-V  PM6:PYFO-V | 1.559  1.556  1.546 | 0.999  0.991  0.968 | | 0.560  0.565  0.578 | 0.271  0.270  0.269 | 0.077  0.085  0.078 | 0.212  0.210  0.231 |

The energy losses of the OSCs were evaluated based on the equation below:

*E*_loss_ = *E*_g_ – *qV*_OC_ = *ΔE*_1_ + *ΔE*_2_ + *ΔE*_3_

where *q* is the elementary charge, *ΔE*_1_ is the radiative energy loss above the bandgap, *ΔE*_2_ is the radiative energy loss below the bandgap, and *ΔE*_3_ is the non-radiative energy loss.

Table. S6 *J*_ph_ vs *V*_eff_ relationship derived parameters.

| Active layer | *J*_sat_ (mA cm^-2^) | | *J*_SC_ (mA cm^-2^) | *J*_MPP_ (mA cm^-2^) | *η*_diss_ (%) | | *η*_coll_ (%) | |
| --- | --- | --- | --- | --- | --- | --- | --- | --- |
| PM6:OBO-2F | | 15.1 | 14.8 | 12.5 | 98.0 | 82.5 | |  |
| PM6:DY4FO-V  PM6:DY6FO-V  PM6:PYFO-V | | 20.2  22.2  22.7 | 20.1  22.1  22.4 | 17.6  19.8  19.1 | 99.4  99.5  98.7 | 87.1  89.2  84.2 | |  |

Table. S7 GISAXS fitting results.

| Active layer | *ξ* (nm) | *η* (nm) | D | 2*R*_g_ (nm) |
| --- | --- | --- | --- | --- |
| PM6:OBO-2F | 29.6 | 7.8 | 2.6 | 33.8 |
| PM6:DY4FO-V  PM6:DY6FO-V  PM6:PYFO-V | 22.3  22.4  21.6 | 6.9  6.5  6.7 | 2.4  2.4  2.3 | 27.9  26.3  26.1 |

References

[1] H. Fu, Q. Wang, Q. Chen, Y. Zhang, S. Meng, L. Xue, C. Zhang, Y. Yi, Z.-G. Zhang, *Angew. Chem. Int. Ed.* 2024, *63*, e202403005.

[2] H. Fu, M. Zhang, Y. Zhang, Q. Wang, Z. Xu, Q. Zhou, Z. Li, Y. Bai, Y. Li, Z.-G. Zhang, *Angew. Chem. Int. Ed.* 2023, *62*, e202306303.

[3] B. Zou, H. M. Ng, H. Yu, P. Ding, J. Yao, D. Chen, S. H. Pun, H. Hu, K. Ding, R. Ma, M. Qammar, W. Liu, W. Wu, J. Y. L. Lai, C. Zhao, M. Pan, L. Guo, J. E. Halpert, H. Ade, G. Li, H. Yan, *Adv. Mater.* *n/a*, 2405404.

[4] J. Liang, M. Pan, Z. Wang, J. Zhang, F. Bai, R. Ma, L. Ding, Y. Chen, X. Li, H. Ade, H. Yan, *Chem. Mater.* 2022, *34*, 2059.

[5] S. L. Mayo, B. D. Olafson, W. A. Goddard, *J. Phys. Chem.* 1990, *94*, 8897.

[6] J. Gasteiger, M. Marsili, *Tetrahedron* 1980, *36*, 3219.

[7] C. Bannwarth, S. Ehlert, S. Grimme, *J. Chem. Theory Comput.* 2019, *15*, 1652.

[8] S. Grimme, S. Ehrlich, L. Goerigk, *J. Comput. Chem.* 2011, *32*, 1456.

[9] T. Lu, F. Chen, *J. Comput. Chem.* 2012, *33*, 580.

[10] B. Zou, W. Wu, T. A. Dela Peña, R. Ma, Y. Luo, Y. Hai, X. Xie, M. Li, Z. Luo, J. Wu, C. Yang, G. Li, H. Yan, *Nano-Micro Lett.* 2024, *16*, 30.

[11] W. Wu, B. Zou, R. Ma, J. Yao, C. Li, Z. Luo, B. Xie, M. Qammar, T. A. Dela Peña, M. Li, J. Wu, C. Yang, Q. Fan, W. Ma, G. Li, H. Yan, *Small* *n/a*, 2402793.

[12] R. Ma, H. Li, T. A. Dela Peña, H. Wang, C. Yan, P. Cheng, J. Wu, G. Li, *Natl. Sci. Rev.* 2024, *11*, nwae384.

[13] S. Luo, F. Bai, J. Zhang, H. Zhao, I. Angunawela, X. Zou, X. Li, Z. Luo, K. Feng, H. Yu, K. S. Wong, H. Ade, W. Ma, H. Yan, *Nano Energy* 2022, *98*, 107281.

[14] F. Bai, J. Zhang, A. Zeng, H. Zhao, K. Duan, H. Yu, K. Cheng, G. Chai, Y. Chen, J. Liang, W. Ma, H. Yan, *Joule* 2021, *5*, 1231.

[15] W. Liu, W. Wu, A. A. Sergeev, J. Yao, Y. Fu, C. H. Kwok, H. M. Ng, C. Li, X. Li, S. H. Pun, H. Hu, X. Lu, K. S. Wong, Y. Li, H. Yan, H. Yu, *Adv. Sci.* *n/a*, 2410826.

[16] H. Gao, B. Fan, L. Yu, Y. Wang, R. Li, W. Jiang, T. Chen, J. Zeng, F. R. Lin, B. Kan, H. Li, L. Wang, A. K.-Y. Jen, *ACS Energy Lett.* 2024, 5541.

[17] M. Zhang, Z. Wang, L. Zhu, R. Zeng, X. Xue, S. Liu, J. Yan, Z. Yang, W. Zhong, G. Zhou, L. Kan, J. Xu, A. Zhang, J. Deng, Z. Zhou, J. Song, H. Jing, S. Xu, Y. Zhang, F. Liu, *Adv. Mater.* *n/a*, 2407297.

[18] X. Yang, Y. Gao, L.-Y. Xu, X. Wu, X. Chen, Y. Shao, B. Xiao, S. Liu, J. Xia, R. Sun, J. Min, *Energy Environ. Sci.* 2024, *17*, 5962.

[19] J. Shi, P. Ding, J. Zhu, Z. Chen, S. Gao, X. Yu, X. Liao, Q. Liu, Z. Ge, *Energy Environ. Sci.* 2024.

[20] Y. Li, L. Mei, Z. Ge, C. Liu, J. Song, Y. Man, J. Gao, J. Zhang, Z. Tang, X.-K. Chen, Y. Sun, *Adv. Mater.* 2024, *36*, 2403890.

[21] Y. Li, Z. Ge, L. Mei, H. Ma, Y. Chen, X. Wang, J. Yu, G. Lu, R. Yang, X.-K. Chen, S. Yin, Y. Sun, *Angew. Chem.* *n/a*, e202411044.

[22] H. Fu, M. Zhang, Y. Zhang, Q. Wang, Z. Xu, Q. Zhou, Z. Li, Y. Bai, Y. Li, Z.-G. Zhang, *Angew. Chem. Int. Ed.* 2023, *62*, e202306303.

[23] X. Gu, Y. Wei, N. Yu, J. Qiao, Z. Han, Q. Lin, X. Han, J. Gao, C. Li, J. Zhang, X. Hao, Z. Wei, Z. Tang, Y. Cai, X. Zhang, H. Huang, *CCS Chem.* 2023, *5*, 2576.

[24] J. Wu, Z. Ling, L. R. Franco, S. Y. Jeong, Z. Genene, J. Mena, S. Chen, C. Chen, C. M. Araujo, C. F. N. Marchiori, J. Kimpel, X. Chang, F. H. Isikgor, Q. Chen, H. Faber, Y. Han, F. Laquai, M. Zhang, H. Y. Woo, D. Yu, T. D. Anthopoulos, E. Wang, *Angew. Chem. Int. Ed.* 2023, *62*, e202302888.

[25] C. Sun, J.-W. Lee, C. Lee, D. Lee, S. Cho, S.-K. Kwon, B. J. Kim, Y.-H. Kim, *Joule* 2023, *7*, 416.

[26] M. Lv, Q. Wang, J. Zhang, Y. Wang, Z.-G. Zhang, T. Wang, H. Zhang, K. Lu, Z. Wei, D. Deng, *Adv. Mater.* 2024, *36*, 2310046.

[27] C. Sun, J.-W. Lee, Z. Tan, T. N.-L. Phan, D. Han, H.-G. Lee, S. Lee, S.-K. Kwon, B. J. Kim, Y.-H. Kim, *Adv. Energy Mater.* 2023, *13*, 2301283.

[28] H. Wang, C. Cao, H. Chen, H. Lai, C. Ke, Y. Zhu, H. Li, F. He, *Angew. Chem.* 2022, *134*, e202201844.

[29] L. Zhang, Z. Zhang, D. Deng, H. Zhou, J. Zhang, Z. Wei, *Adv. Sci.* 2022, *9*, 2202513.

[30] W. Liu, H. Zhang, S. Liang, T. Wang, S. He, Y. Hu, R. Zhang, H. Ning, J. Ren, A. Bakulin, F. Gao, J. Yuan, Y. Zou, *Angew. Chem.* 2023, *135*, e202311645.

[31] F. Yi, M. Xiao, Y. Meng, H. Bai, W. Su, W. Gao, Z.-F. Yao, G. Qi, Z. Liang, C. Jin, L. Tang, R. Zhang, L. Yan, Y. Liu, W. Zhu, W. Ma, Q. Fan, *Angew. Chem. Int. Ed.* 2024, *63*, e202319295.

[32] X. Shen, X. Lai, H. Lai, Y. Wang, H. Li, M. Ou, F. He, *Adv. Funct. Mater.* *n/a*, 2404919.

[33] W. Liu, J. Yuan, C. Zhu, Q. Wei, S. Liang, H. Zhang, G. Zheng, Y. Hu, L. Meng, F. Gao, Y. Li, Y. Zou, *Sci. China Chem.* 2022, *65*, 1374.

[34] P. Tan, H. Chen, H. Wang, X. Lai, Y. Zhu, X. Shen, M. Pu, H. Lai, S. Zhang, W. Ma, F. He, *Adv. Funct. Mater.* 2024, *34*, 2305608.

[35] Y. Liang, D. Zhang, Z. Wu, T. Jia, L. Lüer, H. Tang, L. Hong, J. Zhang, K. Zhang, C. J. Brabec, N. Li, F. Huang, *Nat. Energy* 2022, *7*, 1180.

[36] H. Zhuo, X. Li, J. Zhang, S. Qin, J. Guo, R. Zhou, X. Jiang, X. Wu, Z. Chen, J. Li, L. Meng, Y. Li, *Angew. Chem. Int. Ed.* 2023, *62*, e202303551.

[37] H. Jeon, K. Hong, J.-W. Lee, D. Jeong, T. N.-L. Phan, H.-G. Lee, J. S. Park, C. Wang, S. Xuyao, Y.-H. Kim, B. J. Kim, *Chem. Mater.* 2023, *35*, 9276.

[38] W. Wang, Y. Cui, T. Zhang, P. Bi, J. Wang, S. Yang, J. Wang, S. Zhang, J. Hou, *Joule* 2023, *7*, 1067.

[39] C. Lee, J.-H. Lee, H. H. Lee, M. Nam, D.-H. Ko, *Adv. Energy Mater.* 2022, *12*, 2200275.

[40] S. Xu, H. Wang, R. Ma, J. Huang, Y. Xu, P. Peng, T. Ma, N. Ye, B. Wang, N. Ma, Y. Zhang, W. Gao, X. Hu, G. Li, Y. Chen, *Mater. Sci. Eng. R Rep.* 2025, *166*, 101066.

[41] F. Bai, J. Zhang, A. Zeng, H. Zhao, K. Duan, H. Yu, K. Cheng, G. Chai, Y. Chen, J. Liang, W. Ma, H. Yan, *Joule* 2021, *5*, 1231.

[42] P. Bi, C. An, T. Zhang, Z. Chen, Y. Xu, Y. Cui, J. Wang, J. Li, Y. Wang, J. Ren, X.-T. Hao, S. Zhang, J. Hou, *J. Mater. Chem. A* 2022.

[43] Y. Cui, Y. Wang, J. Bergqvist, H. Yao, Y. Xu, B. Gao, C. Yang, S. Zhang, O. Inganäs, F. Gao, J. Hou, *Nat. Energy* 2019, *4*, 768.

[44] L. Xie, J. Zhang, W. Song, J. Ge, D. Li, R. Zhou, J. Zhang, X. Zhang, D. Yang, B. Tang, T. Wu, Z. Ge, *Nano Energy* 2022, *99*, 107414.

[45] L.-K. Ma, Y. Chen, P. C. Y. Chow, G. Zhang, J. Huang, C. Ma, J. Zhang, H. Yin, A. M. Hong Cheung, K. S. Wong, S. K. So, H. Yan, *Joule* 2020, *4*, 1486.

[46] S. V. Dayneko, M. Pahlevani, G. C. Welch, *ACS Appl. Mater. Interfaces* 2019, *11*, 46017.
